# Conservative and Minimally Invasive Treatments for Temporomandibular Disorders Versus Inactive Controls: A Systematic Review and Meta-Analysis of Randomized Trials

**DOI:** 10.3390/medsci14060660

**Published:** 2026-10-10

**Authors:** Oana Țănculescu, Eugenia-Larisa Țarevici, Petronela Bodnar, Alice-Teodora Chiriac, Marina Cristina Iuliana Iordache, Adrian Doloca, Vlad-Ștefan Proca, Monica Silvia Tatarciuc, Alice-Arina Pendefunda, Georgeta Liliana Foia, Cătălina Iulia Săveanu, Valeriu Fala, Olga Cheptănaru, Gheorghe Bordeniuc, Alina-Mihaela Apostu

**Affiliations:** 1 Grigore T. Popa University of Medicine and Pharmacy Iasi, 16 Universitatii Street, 700115 Iasi, Romania; oana.tanculescu@umfiasi.ro (O.Ț.); eugenia-tarevici@umfiasi.ro (E.-L.Ț.); marina.iordache@umfiasi.ro (M.C.I.I.); adrian.doloca@umfiasi.ro (A.D.); vlad-stefan-proca@umfiasi.ro (V.-Ș.P.); monica.tatarciuc@umfiasi.ro (M.S.T.); alice.ciocan@umfiasi.ro (A.-A.P.); georgeta.foia@umfiasi.ro (G.L.F.); catalina.saveanu@umfiasi.ro (C.I.S.); apostu.alina@umfiasi.ro (A.-M.A.); 2 Nicolae Testemițeanu State University of Medicine and Pharmacy, Stefan Cel Mare Boulevard, 165, MD-2004 Chișinău, Moldova; valeriu.fala@usmf.md (V.F.); olga.cheptanaru@usmf.md (O.C.); gheorghe.bordeniuc@usmf.md (G.B.)

**Keywords:** temporomandibular disorders, rehabilitation medicine, musculoskeletal health, physical therapy, therapeutic exercise, conservative treatment, minimally invasive interventions, mandibular function, systematic review, meta-analysis

## Abstract

**Background/Objectives:** Temporomandibular disorders (TMDs) are musculoskeletal conditions in which rehabilitation aims to reduce pain and restore mandibular mobility and function. We estimated the between-group effects of conservative and minimally invasive treatments versus inactive controls and examined important sources of variation. **Methods:** We included randomized controlled trials published in 2015–2025 in adults diagnosed with RDC/TMD or DC/TMD. PubMed/MEDLINE, Embase, Web of Science Core Collection, Scopus and CENTRAL were searched, with the final searches completed in January 2026. The review was prospectively registered in PROSPERO (CRD420251250251). We synthesized comparisons of conservative and minimally invasive treatments with inactive controls in adults with TMD. Random-effects meta-analyses used REML and modified Hartung–Knapp confidence intervals. Two human reviewers independently assessed risk of bias with RoB 2 and certainty with GRADE. **Results:** Thirty-five studies contributed to 69 effects. Compared with inactive controls, short-term pain was 1.31 points lower on a 0–10 scale (95% CI 0.62–2.00; 23 studies; very-low certainty) and 0.94 points lower (95% CI 0.15–1.74) after high-risk results were excluded. Mouth opening was 1.96 mm greater (95% CI 0.33–3.60; 26 effects from 25 studies; very-low certainty) and 0.75 mm greater (95% CI −0.62 to 2.12) after high-risk results were excluded; the latter confidence interval included no difference. Prediction intervals included no benefit. Evidence for jaw function, oral-health-related quality of life and longer-term effects was sparse. None of the 73 result-specific risk-of-bias assessments was low risk, and 25 were high risk. **Conclusions:** The average short-term pain benefit was modest and decreased after high-risk results were removed. The mouth-opening difference became inconclusive after high-risk results were excluded. These descriptive pooled estimates support transparent discussion of uncertainty in rehabilitation practice but should not be used to rank treatments or predict an individual response.

## 1. Introduction

Temporomandibular disorders (TMDs) are a heterogeneous group of musculoskeletal conditions affecting the masticatory muscles, temporomandibular joints and related structures. For rehabilitation teams, the relevant goals extend beyond pain relief to mandibular mobility, everyday function and self-management within a biopsychosocial model [1,2,3,4]. Prevalence depends on the condition measured: a meta-analysis using RDC/TMD or DC/TMD criteria estimated that temporomandibular joint disorders affect about 31% of adults, including asymptomatic forms such as disk displacement [5]. Persistent orofacial pain also creates substantial healthcare, personal and productivity costs [6,7].

Pain and jaw limitation may coexist with psychological distress and parafunctional behavior, such as tooth clenching or jaw bracing outside normal chewing, swallowing or speaking [1,8].

In clinical rehabilitation, the first-line approach is reversible, conservative and patient-centered. Education and self-management may be combined with therapeutic exercise, manual therapy and, when appropriate, splints or physical-agent modalities. The aims are to reduce pain, restore mandibular mobility and function, and address factors that may maintain symptoms [3,9,10].

For persistent symptoms, clinicians may also consider acupuncture, needling, photobiomodulation, transcutaneous electrical nerve stimulation, occlusal appliances or minimally invasive procedures such as arthrocentesis and injections. These treatments act through different mechanisms and are often used as part of multimodal care [11,12,13,14].

This therapeutic breadth reflects multidisciplinary practice but complicates evidence synthesis. Trials differ in TMD phenotype, treatment dose and duration, comparator credibility, outcome definition and follow-up. A pooled result can therefore hide clinically important differences between the studies it summarizes.

The transition from the Research Diagnostic Criteria for Temporomandibular Disorders (RDC/TMD) to the Diagnostic Criteria for Temporomandibular Disorders (DC/TMD) adds another source of variation. Both systems standardize research diagnosis, but differences in case definition and application can affect comparability [1,2].

Most reviews address one intervention or one TMD subtype. Recent syntheses have examined education and self-management, therapeutic exercise, physiotherapy and occlusal splints [15,16,17,18]. These reviews are clinically useful, but their broader diagnostic criteria and mixture of active and inactive comparators answer different questions from the present review.

The largest synthesis is a network meta-analysis of 153 randomized trials and 8713 participants across 59 interventions [19]. An updated synthesis of diagnosis-defined trials against inactive comparators can clarify treatment–control differences and their uncertainty, while identifying gaps for future randomized trials. Other intervention-specific reviews have examined laser therapy, exercise, splints, botulinum toxin and acupuncture [20,21,22,23,24,25,26].

Active-treatment comparisons estimate relative efficacy between interventions. Inactive controls comprise sham/placebo, waitlist and no-treatment; sham/placebo comparisons seek to control contextual effects, whereas no-treatment and waitlist comparisons include these effects in the treatment–control difference. Restricting the pooled analyses to inactive controls therefore provides a distinct estimate and avoids presenting an active-treatment ranking from these broad pooled estimates.

This review estimated the effects of conservative and minimally invasive treatments on pain, mouth opening, jaw functional limitation and oral-health-related quality of life in adults with RDC/TMD- or DC/TMD-defined TMD, compared only with inactive controls. We also examined result-specific risk of bias, the influence of high-risk results and clinically relevant sources of between-study variation. The review was designed to inform multidisciplinary rehabilitation decisions by describing and explaining the evidence, not by ranking treatments.

## 2. Materials and Methods

### 2.1. Protocol Registration and Reporting Standards

The protocol was published separately [27], and the review was prospectively registered in PROSPERO (CRD420251250251) [28]. The registered review encompassed both inactive and active comparators. The present article reports analyses involving inactive controls (sham/placebo, no treatment or waitlist); this scope restriction is reported in Section 2.8. The review is reported in accordance with PRISMA 2020 [29], the protocol was prepared according to PRISMA-P [30], and reporting was informed by the PRISMA 2020 explanation and elaboration guidance [31].

### 2.2. Eligibility Criteria (PICOS Framework)

Eligibility followed the registered PICOS framework; the refinements made during implementation are summarized in Section 2.8.

Population (P)

Adults aged 18 years or older with muscular, articular or mixed TMD diagnosed using DC/TMD or RDC/TMD were eligible. Studies that did not use either diagnostic framework were excluded, as were studies in which TMD was secondary to systemic inflammatory disease, tumor, recent trauma or recent TMJ surgery.

Intervention (I)

Eligible conservative treatments included education and self-management, exercise, physiotherapy, manual therapy, occlusal appliances, photobiomodulation, TENS, acupuncture and related reversible approaches.

Eligible minimally invasive treatments included dry needling, botulinum toxin, arthrocentesis and intra-articular injections. Open surgery and drug-only treatment were excluded.

For subgroup analyses, treatments were grouped by their main clinical approach: physical/manual therapy; occlusal appliances; physical agents; acupuncture; neuromuscular needling or injection; and joint procedures.

Comparator (C)

Eligible comparators were sham/placebo, no treatment and waitlist controls.

Outcomes (O)

The analyses covered four domains: pain intensity, mouth opening, jaw functional limitation and oral-health-related quality of life. Only these domains were reported often enough and in sufficiently comparable form for quantitative synthesis.

Outcomes were grouped as short term (≤3 months), medium term (>3 to ≤6 months) and long term (>6 months). When several assessments fell within one window, the latest eligible time point was used. Timing uncertainties were examined in sensitivity analyses.

Study Design (S)

English-language, full-text, peer-reviewed randomized controlled trials published in 2015–2025 were eligible. The lower limit was selected because DC/TMD was published in 2014 [1]; studies using RDC/TMD remained eligible.

The eligibility criteria are summarized in Table 1.

### 2.3. Search Strategy

We searched PubMed/MEDLINE, Embase, Web of Science Core Collection, Scopus and Cochrane CENTRAL, then screened reference lists and citing articles. Trial registries were checked for unpublished eligible trials and registration details; no unpublished trial contributed data. The final database searches were completed in January 2026 and were restricted to reports published from 1 January 2015 through 31 December 2025.

Search concepts covered TMD, conservative or minimally invasive treatments, randomized trials and, where applicable, functional or quality-of-life terms. The syntax and limits were adapted for each database. Complete database-specific strategies are provided in Supplementary Table S1.

### 2.4. Study Selection and Data Collection

Two human reviewers independently screened titles, abstracts and full texts in Rayyan [32] and extracted the data; a third reviewer resolved disagreements. Companion reports from the same trial were linked so that each study was counted once.

Values reported in tables were preferred to narrative text. Pain scales were converted to 0–10, standard errors to standard deviations, and medians with interquartile ranges to means and standard deviations only when established methods were applicable [33,34]. Endpoint contrasts were preferred. Of the 69 effects in the overall analyses, 68 used endpoint values and one short-term mouth-opening effect used a reported change score because a compatible endpoint dispersion was unavailable; an exclusion sensitivity assessed its influence.

For each study, we recorded the diagnostic framework and phenotype, treatment content and exposure, comparator, sample size, outcome and analyzed time point. Missing details were recorded as not reported rather than inferred.

One comparison was retained per study, outcome and time window. When several eligible treatment arms shared one inactive control, the active arms were combined so that the control was counted once. For arms j, the combined sample was nc = Σnj, the combined mean was Σ(nj × meanj)/nc, and the combined standard deviation retained both within- and between-arm variation: SDc = √{[Σ(nj − 1)SDj^2^ + Σnj(meanj − meanc)^2^]/(nc − 1)} [35]. Arm-level contrasts were retained only for exploratory treatment-cluster analyses, with shared-control covariance accounted for. Independently randomized and analyzed cohorts with separate placebo groups were retained as separate effects but counted as one study; therefore, short-term mouth opening includes 26 effects from 25 studies. In Aisaiti, the pooled parent effect was replaced by the two independently randomized cohorts because each cohort had its own placebo group.

Comparators were classified according to what participants received before outcome measurement. Sham/placebo controls reproduced the treatment context without the active component; no-treatment and waitlist controls received no outcome-directed treatment.

### 2.5. Comparator and Outcome Definitions

Only inactive contrasts were synthesized. The primary estimand was the descriptive average between-group effect across all eligible interventions versus an inactive control; it was not an intervention-specific effect or a treatment ranking. These comparator conditions were pooled to summarize the broad inactive-control question, with comparator strata examined separately because contextual effects differ. Pain was expressed as the mean difference on a 0–10 scale and mouth opening as the difference in millimeters. Jaw functional limitation and quality of life were expressed as standardized effects when instruments differed. Negative values favor treatment for pain, functional limitation and quality of life; positive values favor treatment for mouth opening. GRADE therefore applies to this broad inactive-control question and does not support treatment-specific recommendations.

### 2.6. Statistical Analysis

Random-effects models were used because the studies differed clinically. Between-study variance was estimated with REML, and confidence intervals used a conservatively modified Hartung–Knapp method [35,36,37,38,39,40]. Hedges g was used when outcomes required standardization [41]. Heterogeneity was described with I^2^ and τ [42], prediction intervals were reported when at least three studies were available [43], and funnel plots and Egger tests were limited to analyses with at least 10 effects [44].

Analyses were conducted using reproducible scripts in R version 4.6.1 (R Foundation for Statistical Computing, Vienna, Austria), with the readxl 1.5.0 and metafor packages (metafor version 5.0–1) [45,46].

For clinical interpretation, we used operational reference values of 1 point for pain, 3.5 mm for mouth opening, 0.20 standardized units for jaw function and quality of life, and 5 OHIP-14 points [47,48,49,50,51,52,53]. The 1-point pain value was used as a pragmatic reference informed by variable TMD change estimates [47,48,53]; the 3.5 mm value derives from patient-level change in pain-free opening in muscular TMD [49]. These values are contextual guides, not validated universal cut-offs for between-group effects, and patient-level change thresholds are not directly transferable to such effects.

Between-study variability is reported as I^2^ and τ. Because τ is expressed in the units of pain or mouth opening, clinicians can compare it directly with the operational threshold. Estimates based on two to four studies were treated as unstable.

Sensitivity analyses excluded high-risk results, graph-extracted values, converted distributions and assessments obtained immediately or within 48 h. Immediate and very early results remained in the primary short-term set because the prespecified short-term window was ≤3 months; their removal distinguished an early procedural response from the effect of a completed treatment course. We also removed each study in turn and tested an alternative phenotype classification. Comparator-stratified analyses and the six-level therapeutic-cluster model were exploratory; phenotype, diagnostic framework and broad intervention category were evaluated when sufficient data were available.

When pooling was not appropriate, findings were summarized descriptively by treatment, comparator and outcome, with emphasis on direction, consistency and clinical relevance.

### 2.7. Risk-of-Bias Assessment and Certainty of Evidence

Two human reviewers independently assessed risk of bias with Cochrane RoB 2 for every result contributing to a pooled analysis [54]. Judgments targeted the effect of assignment to intervention and were specific to the outcome and time window. Disagreements were resolved by a third human reviewer. The same judgments informed the sensitivity analyses and GRADE assessment.

Two human reviewers independently assessed certainty with GRADE for each outcome and follow-up window [55,56]. Disagreements were resolved by a third human reviewer. Evidence was judged across risk of bias, inconsistency, indirectness, imprecision and publication bias. TMD-specific values for pain and mouth opening were used only to contextualize imprecision, not as universal thresholds [48,49].

### 2.8. Protocol Deviations and Analytical Refinements

Implementation refinements were documented against the PROSPERO record and published protocol [27,28]. The decision to restrict this article to inactive-control comparisons was made after deduplication of the search records and before screening and data extraction, considering the volume of records identified and the anticipated length of a report covering both active and inactive comparators. This decision was not recorded as a dated amendment to the PROSPERO record. Active comparator comparisons were intended for a separate report. Diagnostic eligibility was restricted to trials that explicitly used RDC/TMD or DC/TMD. Analyses used REML, modified Hartung–Knapp intervals, τ, prediction intervals and non-overlapping follow-up windows.

## 3. Results

### 3.1. Study Selection

**Figure 1:**
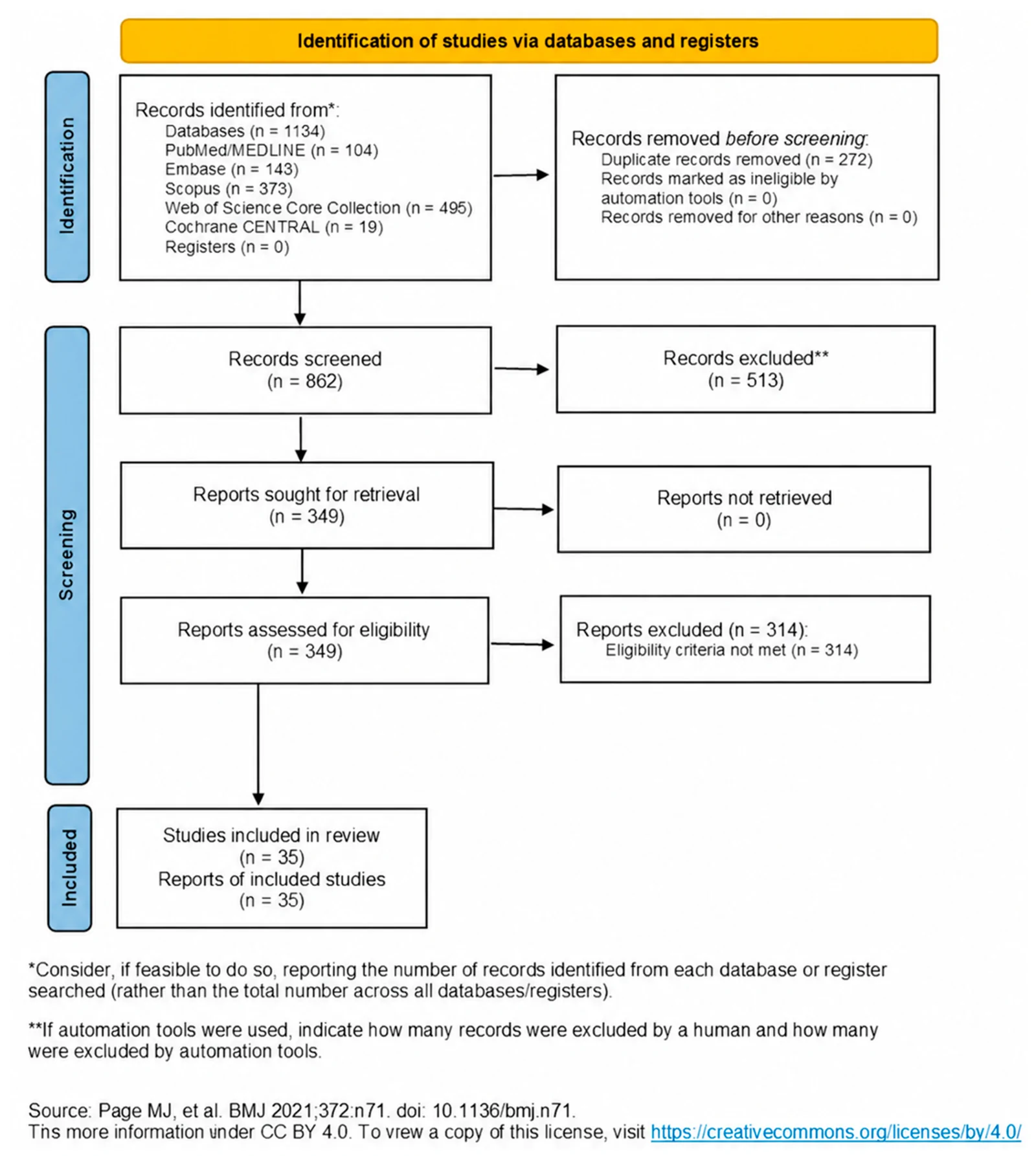
PRISMA 2020 flow diagram of study selection [29].

The database searches identified 1134 records—104 from PubMed/MEDLINE, 143 from Embase, 373 from Scopus, 495 from the Web of Science Core Collection and 19 from Cochrane CENTRAL. Reference lists and citing articles were also checked, but no additional unique records were identified. Trial registries were checked for unpublished eligible trials and registration details; no unpublished trial contributed data. After 272 duplicate records were removed, 862 records underwent title and abstract screening, of which 513 were excluded. All 349 reports sought for retrieval were obtained and assessed for eligibility; 314 did not meet the eligibility criteria, leaving 35 randomized studies included in the review (Figure 1). All 35 contributed to at least one quantitative synthesis. Sixty-nine effects entered the nine overall outcome-by-follow-up analyses. Beyond these overall-analysis effects, four subgroup-only arm-level contrasts—two each from Aroca and Gikić—were used exclusively in the exploratory treatment-cluster analysis. Together, the 69 overall-analysis effects and four subgroup-only contrasts yielded 73 result-specific RoB 2 assessments. Their distribution and judgments are reported in Section 3.4.

### 3.2. Characteristics of Included Studies

Thirty-five unique studies met the final eligibility criteria and contributed to at least one synthesis [57,58,59,60,61,62,63,64,65,66,67,68,69,70,71,72,73,74,75,76,77,78,79,80,81,82,83,84,85,86,87,88,89,90,91]. Together they provided 69 effects across nine outcome and follow-up combinations. Reported analyzed samples ranged from 18 to 145 per study and summed to approximately 1879–1896 participants; the range reflects outcome-specific or inconsistently reported denominators in two trials rather than additional cohorts. The largest bodies of evidence were short-term pain (23 studies; 1221 participants) and short-term mouth opening (26 effects from 25 studies; 1340 participants) (Table 2). Medium- and long-term evidence was much smaller.

The intervention mix was clinically diverse: 12 studies evaluated electrophysical or light-based modalities; 10 evaluated manual therapy, exercise, massage or education; seven evaluated needling or injectable treatments; two evaluated only occlusal splints; two evaluated only acupuncture; and two multi-arm studies spanned intervention classes. Exposure ranged from one immediate session to daily home treatment or continuous splint use over 8 months. Even within photobiomodulation, wavelengths ranged from 635 to 905 nm, application sites varied from specific muscles to the TMJ or patient-defined painful points, and schedules ranged from one to 14 sessions.

Comparator content also differed. Sham devices, thin placebo splints, nonpenetrating acupuncture or saline injections controlled for treatment context more closely than waitlist or no-treatment groups. These clinical differences provide a direct explanation for part of the observed between-study variability and restrict interpretation of the overall pooled estimate to the broad inactive-control question.

In multi-arm trials, only contrasts with an inactive control were included in the analyses. Shared controls were included only once within each analysis.

**Figure 2:**
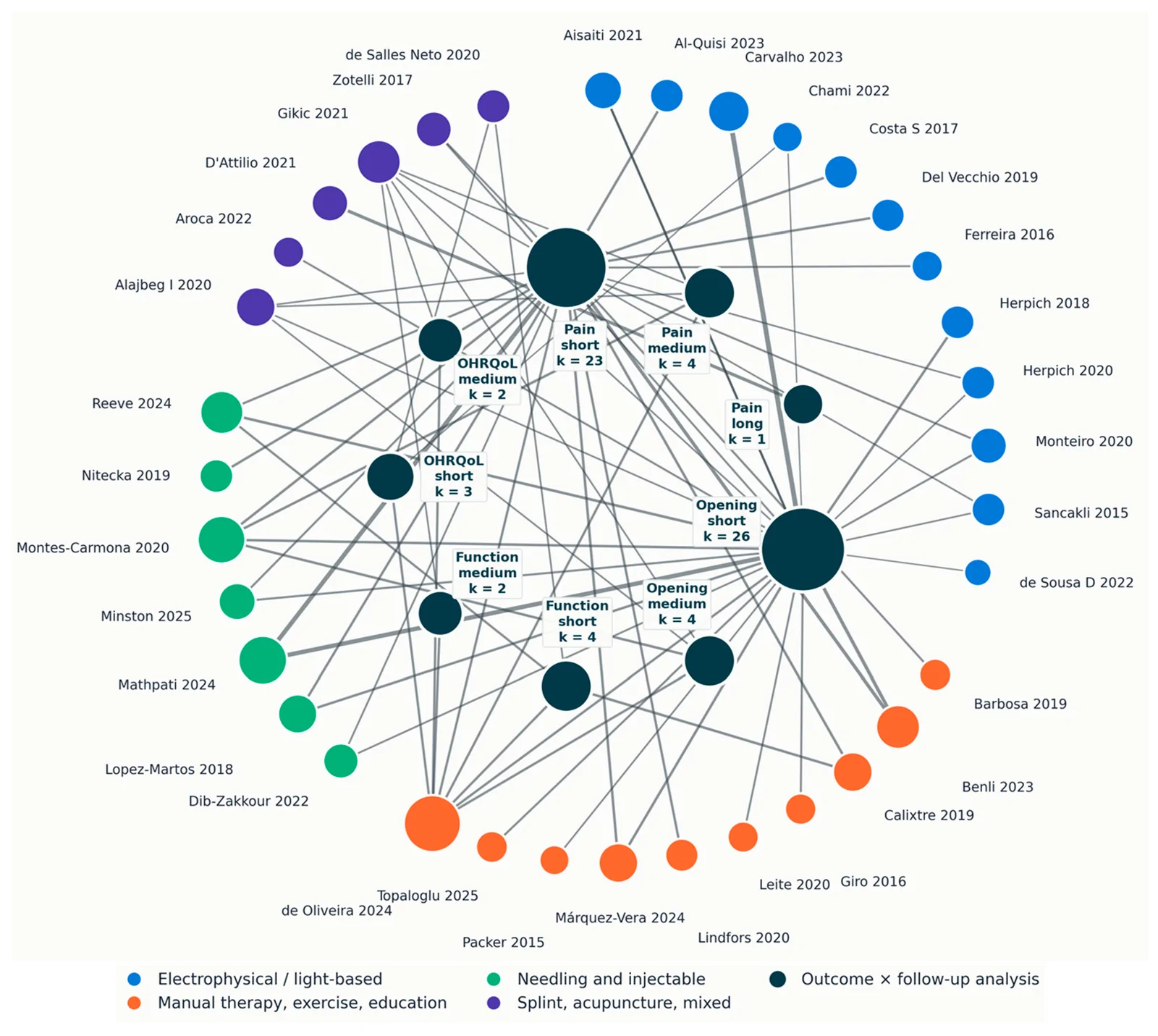
Distribution of evidence across nine outcome-by-follow-up analyses from 35 randomized studies. Each spoke represents one study comparison; central nodes represent analyses and peripheral nodes represent contributing studies. Central-node area reflects the number of effects, peripheral study-node area reflects the analyzed sample, and color indicates treatment family. Most evidence concerns short-term pain and mouth opening.

Figure 2 shows that most evidence concerned short-term pain and mouth opening. Each of the other seven analyses contained only one to four comparisons, which limits treatment-specific interpretation.

### 3.3. Main Quantitative Findings

The nine outcome-by-follow-up analyses are presented below. Negative mean differences favor treatment for pain; positive mean differences favor treatment for mouth opening. The same analytic dataset was used for the main and sensitivity analyses.

Pain and mouth opening provided most of the evidence. Jaw function and oral-health-related quality of life were reported less often and produced sparse, imprecise analyses.

#### 3.3.1. Pain

##### Short-Term Pain

**Figure 3:**
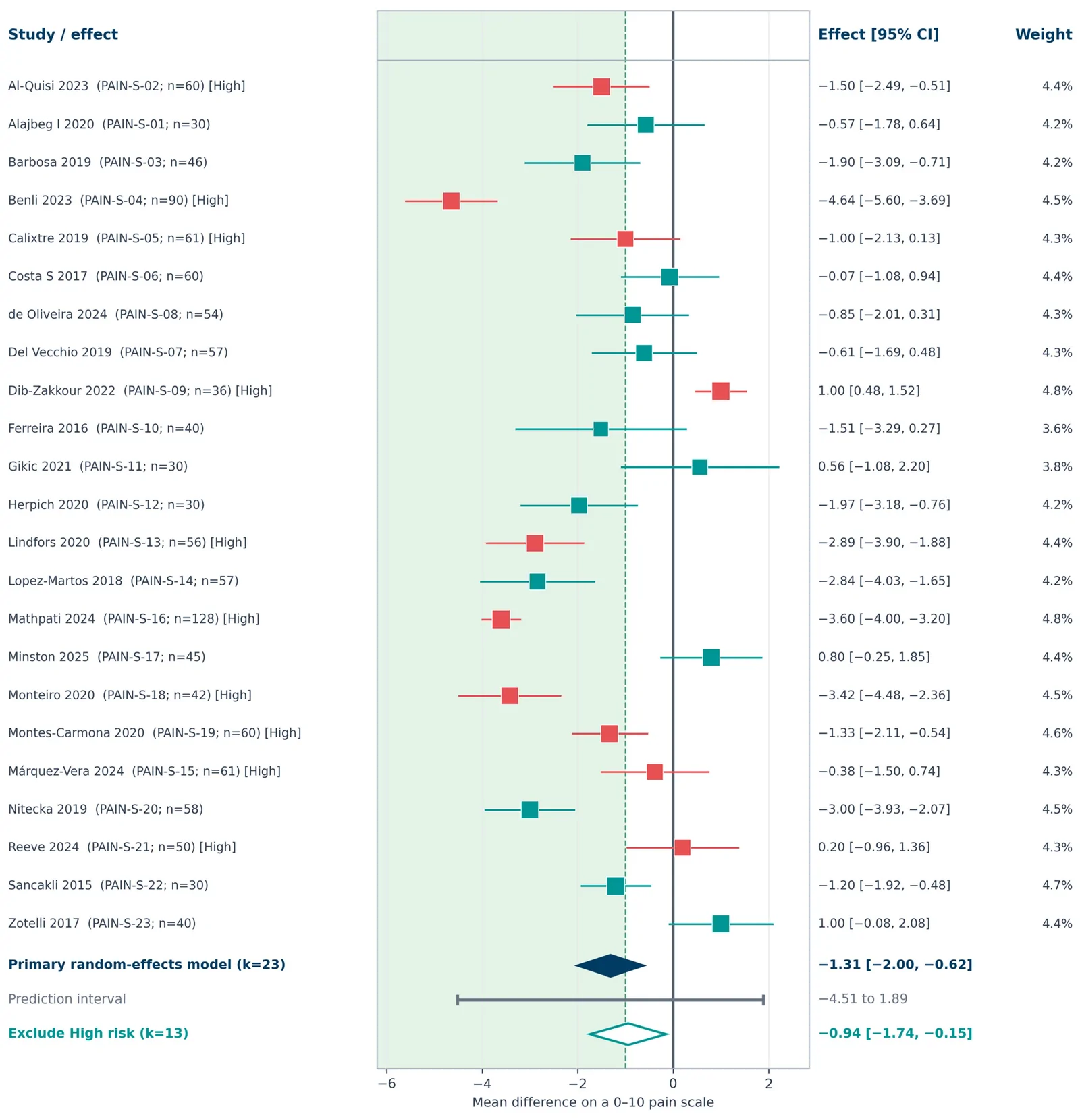
Short-term pain versus inactive controls. The filled diamond shows the main random-effects estimate; the hollow diamond shows the estimate after high-risk results were excluded. The shaded band marks the 1-point operational threshold. Study-level confidence intervals are conventional 95% CIs; pooled confidence intervals use modified Hartung–Knapp inference. The reference value is not a validated universal between-group MCID. Squares represent individual effect estimates, with their area proportional to the random-effects inverse-variance weight; horizontal lines indicate the corresponding 95% confidence intervals.

Across 23 studies, treatment reduced pain by 1.31 points more than control on a 0–10 scale (95% CI 0.62 to 2.00 points better). This average is just above the 1-point threshold used in this review. However, study results differed markedly: τ^2^ was 2.25 and τ was 1.50 points, so the standard deviation of true effects across studies was one and a half times the operational reference value (I^2^ 93.6%). The prediction interval ranged from 4.51 points better to 1.89 points worse. In other words, a future similar setting could show a worthwhile benefit, little difference or no benefit. Certainty was very low (Figure 3).

Ten results were at high risk of bias. Removing them reduced the average benefit to 0.94 points (95% CI 0.15 to 1.74 points better), below the 1-point threshold, and the prediction interval still included no benefit. Read clinically, the mean short-term difference sits close to the operational reference used here, the prediction interval admits both appreciable benefit and none at all, and the fall in the estimate once high-risk results are set aside indicates that study limitations contributed to the apparent magnitude. The main estimate should therefore be read as an upper bound rather than as a typical expectation.

Exploratory comparator-stratified analyses showed an average pain reduction of 1.07 points with sham/placebo controls (k = 20; 95% CI 0.38 to 1.77 points better; τ 1.37; I^2^ 93.5%). With no treatment/waitlist controls, the average difference favored treatment by 2.86 points, but the confidence interval ranged from 1.66 points worse to 7.38 points better (k = 3; τ 1.74; I^2^ 91.5%). The latter estimate was too imprecise for a stable comparator conclusion. Excluding the two graph-derived and two distributionally converted pain effects left 19 studies and an average reduction of 0.96 points (95% CI 0.27 to 1.66 points better), showing a similar but smaller benefit.

##### Medium- and Long-Term Pain

Four studies suggested a 1.12-point medium-term reduction in pain (95% CI 0.23 to 2.02 points better). The prediction interval extended from 2.33 points better to 0.09 points worse, so benefit was not assured across settings. Certainty was low—the highest rating anywhere in this review. When the single high-risk result was removed, the estimate became less precise and included no difference.

Long-term evidence came from one high-risk study of 86 participants that tested a personalized mandibular repositioning appliance [67]. It reported a large difference favoring treatment, but there was no second study to confirm it. The sample was also well short of what a trial would need to detect a 1-point difference: with a pooled standard deviation of 1.93 points, approximately 117 participants would be required at 80% power and 157 at 90%. Certainty was very low, the result should not be generalized to other interventions, and a durable benefit remains unproven.

#### 3.3.2. Mouth Opening

##### Short-Term Opening

**Figure 4:**
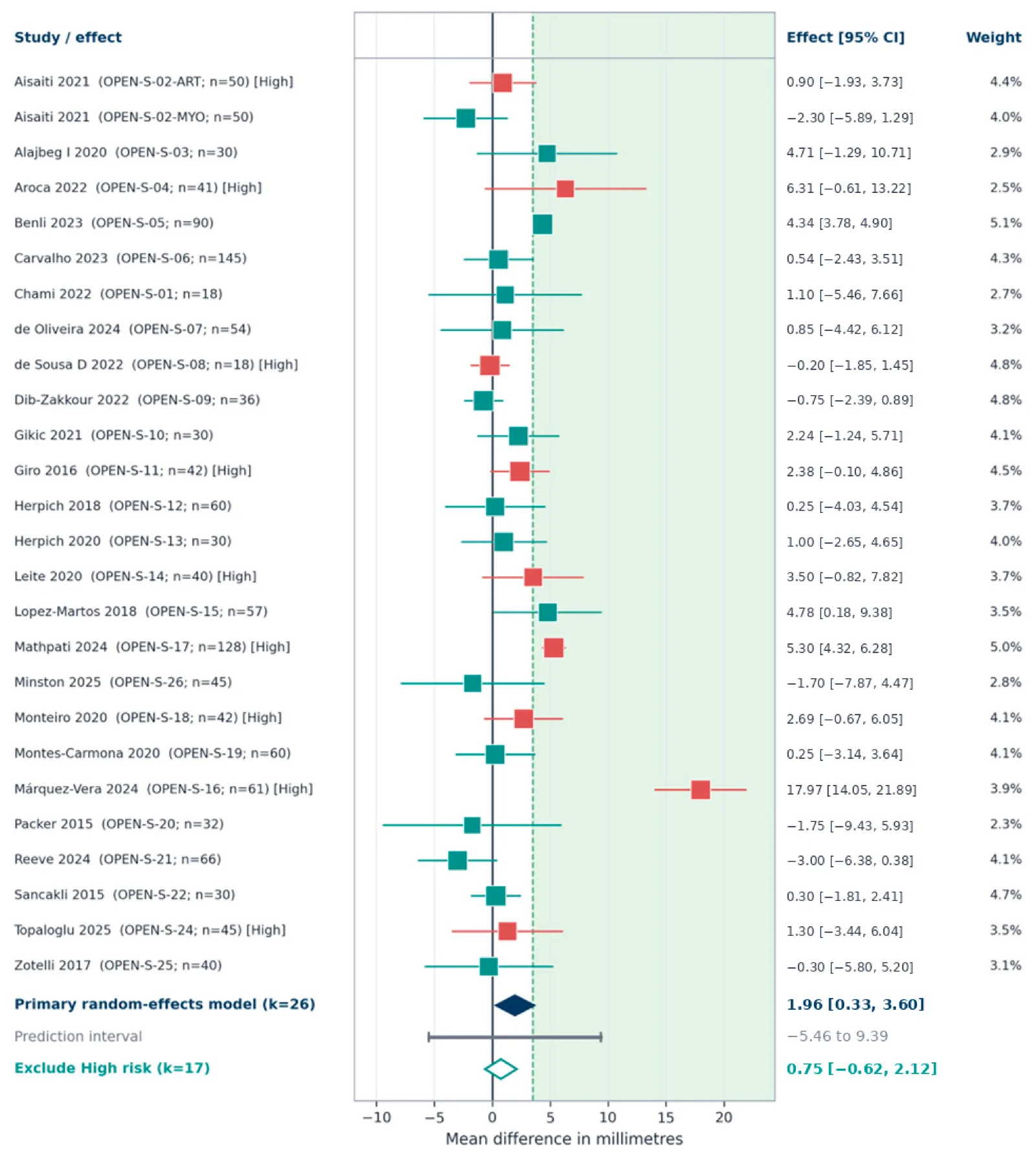
Short-term mouth opening versus inactive controls. The filled diamond shows the main random-effects estimate; the hollow diamond shows the estimate after high-risk results were excluded. The shaded band marks the 3.5 mm operational threshold. Study-level confidence intervals are conventional 95% CIs; pooled confidence intervals use modified Hartung–Knapp inference. The reference value is not a validated universal between-group MCID. Squares represent individual effect estimates, with their area proportional to the random-effects inverse-variance weight; horizontal lines indicate the corresponding 95% confidence intervals.

Across 26 effects from 25 studies, mouth opening increased by 1.96 mm more with treatment than with control (95% CI 0.33 to 3.60 mm). The average was below the 3.5 mm threshold used in this review. Results were inconsistent to an unusual degree: τ^2^ was 12.32 and τ was 3.51 mm (I^2^ 85.9%), so the standard deviation of true effects across studies was equal to the operational reference value. The prediction interval ran from 5.46 mm worse to 9.39 mm better. Certainty was very low (Figure 4).

After nine high-risk results were removed, the average increase was 0.75 mm (95% CI 0.62 mm worse to 2.12 mm better), so this analysis no longer showed a clear advantage. Exploratory comparator strata were also inconclusive: sham/placebo controls yielded k = 23, MD 1.84 mm (95% CI −0.04 to 3.71; τ 3.79 mm; I^2^ 85.5%), and no treatment/waitlist yielded k = 3, MD 3.43 mm (95% CI −0.46 to 7.33; τ 1.13 mm; I^2^ 46.6%). Excluding the Aroca comparison, which combined auricular acupuncture and an occlusal splint for the broad overall estimand, left k = 25 and MD 1.85 mm (95% CI 0.18 to 3.52), indicating that this combined intervention did not determine the result. Clinically, the average gain did not reach the operational threshold and became statistically inconclusive when high-risk results were removed. The pooled measurements also combine pain-free, maximum unassisted and assisted opening, which are related but not interchangeable constructs.

##### Medium-Term Opening

Four studies reported medium-term opening. The average difference was 2.25 mm greater with treatment, but the 95% CI ranged from 1.60 mm worse to 6.10 mm better. The result therefore did not establish a benefit, and certainty was very low. Between-study variance was small in this analysis (τ 0.81 mm, I^2^ 14.2%), but at four studies this should not be read as evidence of consistency.

#### 3.3.3. Jaw Function and Oral-Health-Related Quality of Life

For jaw functional limitation, four short-term studies and two medium-term studies produced small average standardized effects, but both confidence intervals included no difference. In Reeve, the JFLS-8 table title described the data as medians, whereas the table footnote identified them as means and standard deviations; the registry reported medians and interquartile ranges. The analysis followed the summary-statistic designation provided in the article footnote. Excluding this study did not change the conclusion (g = −0.46, 95% CI −1.22 to 0.29; three studies) [88].

Quality-of-life evidence was similarly sparse. Three short-term studies showed no clear standardized benefit, and two medium-term OHIP-14 studies produced an extremely imprecise estimate. Certainty for every function and quality-of-life analysis was very low. Current evidence therefore cannot confirm that improvements in pain or opening reliably translate into better daily function or oral-health-related quality of life.

The two medium-term analyses each contained only two studies. Their confidence intervals are calculable but too unstable for clinical interpretation. For jaw function the estimate was g −0.24 (95% CI −3.20 to 2.71), and for oral-health-related quality of life it was an estimated 2.29-point improvement in OHIP-14 points (95% CI −31.30 to 35.89)—an interval spanning 67 points on an instrument whose full range is 0 to 56. The extreme width follows from modified Hartung–Knapp inference with one degree of freedom. Both rows are retained for completeness and indicate an absence of usable evidence.

#### 3.3.4. Clinical Interpretation Against Operational Thresholds

**Figure 5:**
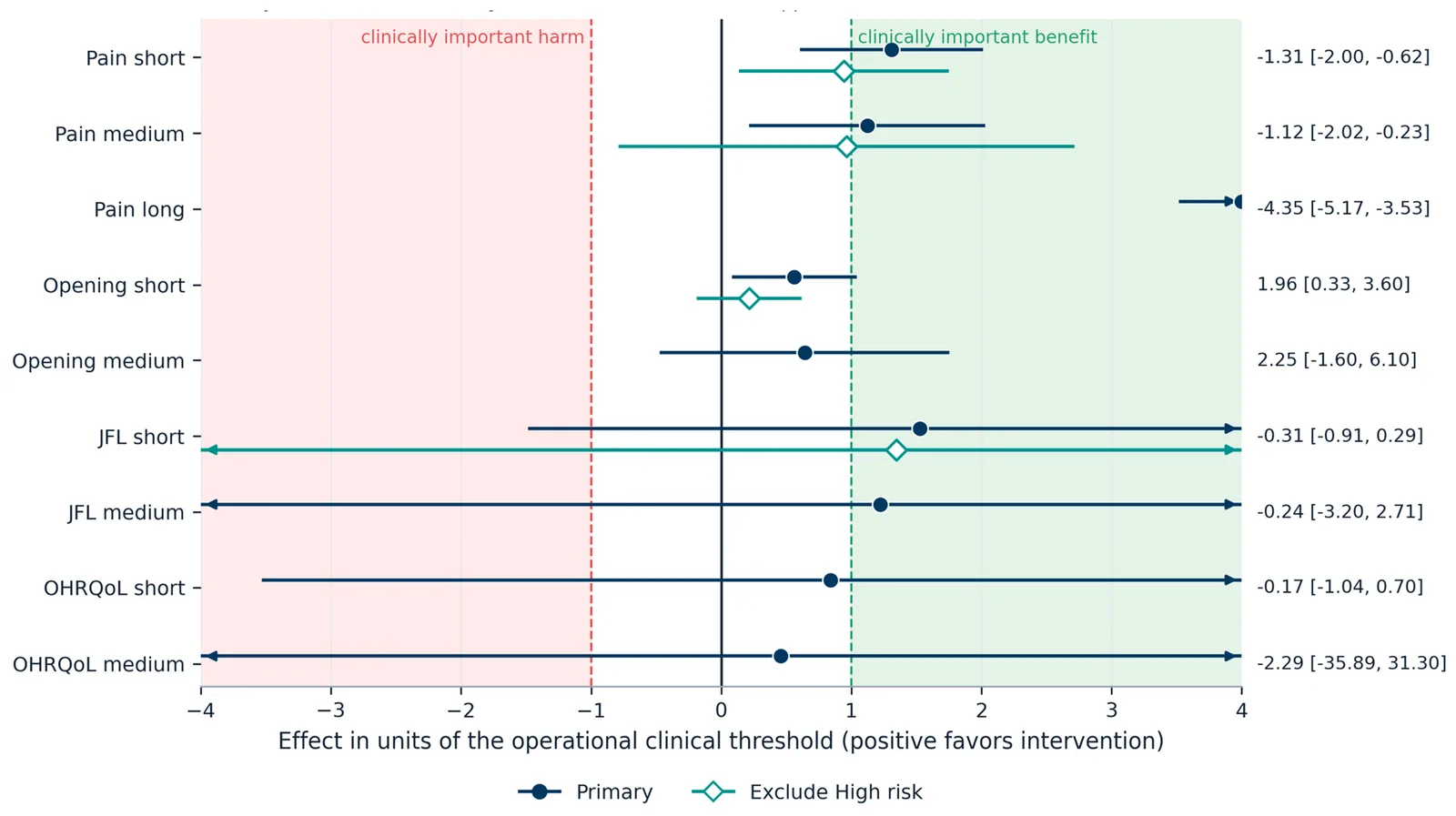
Pooled effects expressed relative to the operational clinical threshold. A magnitude of 1 reaches the threshold; arrows indicate confidence intervals extending beyond the plotted range. Thresholds were pain 1 point on a 0–10 scale, mouth opening 3.5 mm, jaw functional limitation and short-term OHRQoL 0.20 Hedges g, and medium-term OHRQoL 5 OHIP-14 points. These are interpretive thresholds, not universally validated between-group minimal important differences. Squares represent individual effect estimates, with their area proportional to the random-effects inverse-variance weight; horizontal lines indicate the corresponding 95% confidence intervals.

Figure 5 places each pooled result against its operational clinical threshold. A value of 1 reaches the threshold; values below 1 are smaller. This display separates statistical significance from potential clinical importance.

#### 3.3.5. Between-Study Variation and Follow-Up Timing

Between-study variation was large and, for the two main analyses, similar to the operational clinical thresholds (Table 3). For short-term pain, τ was 1.50 points; for short-term mouth opening, τ was 3.51 mm. Estimates from the smaller analyses were unstable, so a τ^2^ value of zero should not be interpreted as evidence that the studies were homogeneous.

Part of the variation reflected measurement timing rather than treatment. Selected short-term assessments ranged from immediately after one procedure to three months of treatment. Excluding four confirmed immediate or ≤48 h opening measurements and the Packer assessment at 2–4 days left 21 effects and reduced the pooled gain from 1.96 to 1.69 mm (95% CI 0.47 to 2.92), τ from 3.51 to 1.97 mm and I^2^ from 85.9% to 78.3%. Removing only the immediate result with the largest gain left 25 effects and reduced the estimate to 1.39 mm (95% CI 0.30 to 2.48; τ 1.91 mm; I^2^ 80.5%). For pain, excluding the four measurements obtained immediately or within 48 h—Costa, Dib-Zakkour, Ferreira and Márquez-Vera—left 19 studies and increased the average reduction from 1.31 to 1.55 points (95% CI 0.78 to 2.32; τ 1.50; I^2^ 91.5%). Source-format checks were similarly informative: excluding two graph-derived and two distributionally converted pain effects gave 0.96 points better (95% CI 0.27 to 1.66; 19 studies), while excluding two converted opening effects gave 1.28 mm greater (95% CI 0.17 to 2.39; 24 effects). Excluding the single change-score opening effect left 25 effects and a nearly unchanged estimate of 1.95 mm (95% CI 0.23 to 3.66). Immediate responses therefore influenced pain and opening differently and should not be interpreted as the effect of a completed treatment course.

#### 3.3.6. Subgroup and Moderator Analyses

Short-term pain improved more in trials restricted to myogenous TMD (10 effects; 523 participants; 2.16 points, 95% CI 1.31 to 3.01) than in mixed-phenotype trials (11 effects; 513 participants; 0.33 points, 95% CI 0.50 points worse to 1.16 points better). The interaction was Q = 10.42 (*p* = 0.001); modified Hartung–Knapp inference gave *p* = 0.004. Two effects with 185 participants could not be classified. Leave-one-out *p* values ranged from <0.001 to 0.010, but under the alternative recruitment-target phenotype rule the interaction weakened (*p* = 0.073). The finding is therefore a classification-sensitive, study-level hypothesis rather than a treatment-selection rule.

The remaining moderator analyses did not explain pain heterogeneity: diagnostic framework (modified Hartung–Knapp *p* = 0.615), conservative versus minimally invasive treatment (*p* = 0.484), and the exploratory six-level therapeutic cluster (*p* = 0.108; correlation sensitivities *p* = 0.106–0.110). The six treatment clusters contained only 1–7 contrasts and still grouped clinically different protocols. Their separate point estimates were therefore considered too unstable and potentially misleading to present as treatment-family effects; the model is reported only as an exploratory interaction test. The outcome-by-follow-up syntheses in Table 4 were prespecified, although several contained too few studies to provide precise estimates. Egger tests did not indicate asymmetry for pain (primary *p* = 0.256; non-high-risk subset *p* = 0.734). For mouth opening, the primary test was borderline (*p* = 0.052) and the non-high-risk subset was asymmetric (*p* = 0.002). Because opening constructs and comparator types also varied with study size, this signal was treated as unresolved rather than as proof of publication bias.

### 3.4. Risk of Bias

**Figure 6:**
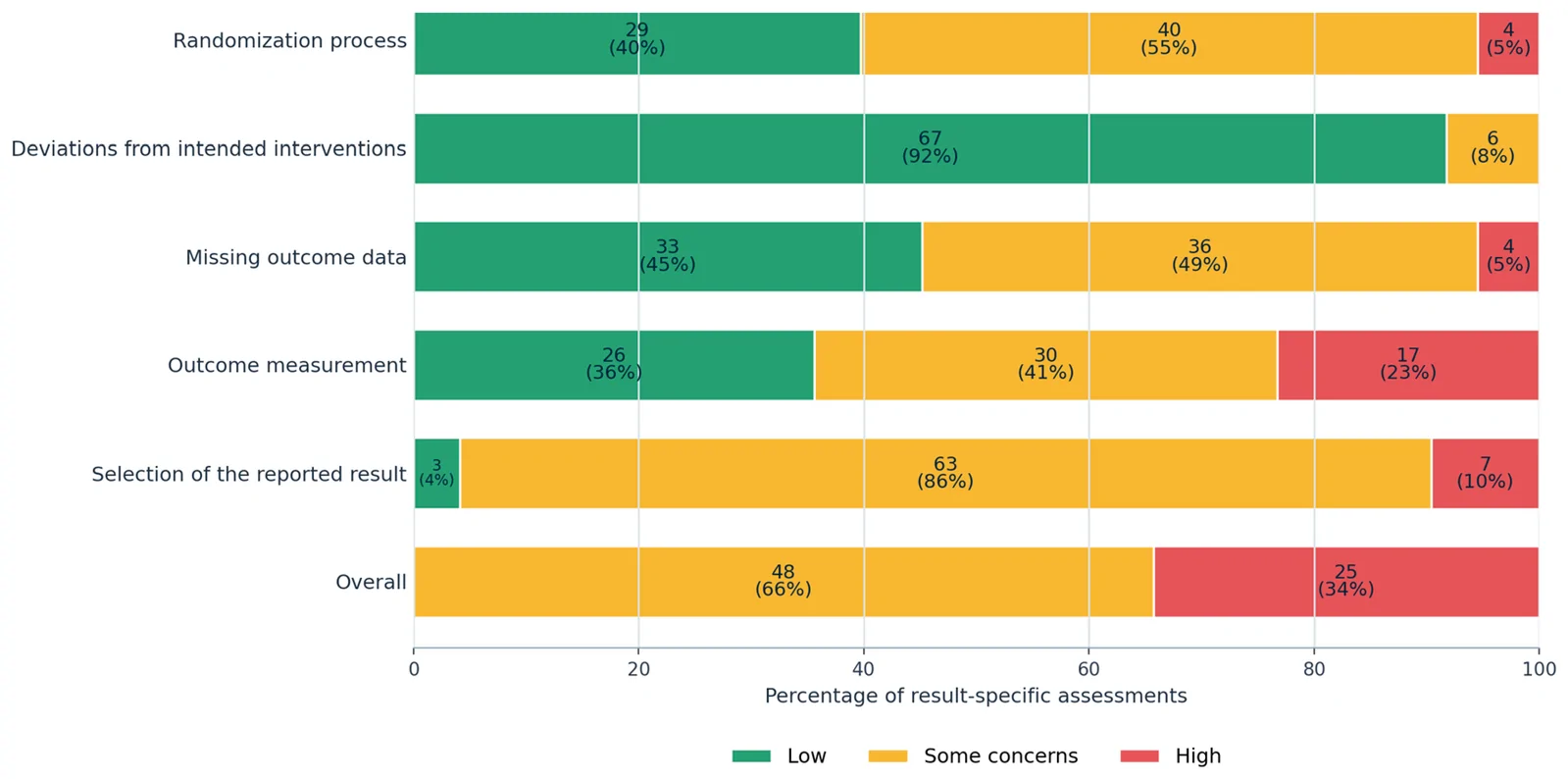
Final RoB 2 judgments for the 73 result-specific assessments from 35 randomized studies. Sixty-nine assessments correspond to effects in the overall outcome-by-follow-up analyses; four additional arm-level contrasts were used only in the exploratory treatment-cluster model. Final RoB 2 judgments target the effect of assignment to intervention. Percentages refer to assessments, not unique studies.

Risk of bias was assessed for 73 active result-specific contrasts from the 35 randomized studies because one study could contribute outcomes, time points or arm-level subgroup contrasts with different limitations. None was low risk overall: 48 raised some concerns and 25 were high risk. Before adjudication, the two human reviewers agreed on the overall rating for 45 of 73 assessments (61.6%); 24 assessments (32.9%) were concordant across all five domains and the overall judgment. All disagreements were resolved by a third human reviewer. The most common problems involved unavailable analysis plans, incomplete outcome data and unclear outcome measurement (Figure 6). Sensitivity analyses excluding high-risk results are therefore central to interpretation.

Registry checks identified mismatches between registered and reported study details, outcomes and participant flow. Because these discrepancies could affect outcome selection or the handling of missing data, they were incorporated into the risk-of-bias judgments.

### 3.5. Certainty of Evidence

Certainty was rated with GRADE for each of the nine broad outcome-by-follow-up questions, not for individual treatments. Eight were rated very low and one—medium-term pain—low. Every analysis was downgraded for risk of bias, because no result in this corpus was at low risk overall, and every analysis was downgraded for imprecision: seriously in five analyses and very seriously in four analyses based on only two to four studies. Inconsistency removed a further two levels from each of the two principal analyses, where the between-study standard deviation matched or exceeded the operational reference value, and one level from short-term jaw function. Mouth opening was additionally downgraded for indirectness at both short and medium term, because pain-free, maximum unassisted and assisted opening were combined. No analysis was downgraded for publication bias: the asymmetry seen in the non-high-risk mouth-opening subset was not present clearly in the full pool, and clinical and construct heterogeneity offered plausible alternative explanations. These ratings characterize the broad inactive-control evidence and must not be transferred to a specific treatment recommendation.

Table 5 summarizes the seven main efficacy outcomes. Three readings deserve emphasis. The single low-certainty result—a 1.12-point reduction in pain between three and six months, from four studies—is the strongest statement this evidence base supports, and it too became inconclusive once the one high-risk result was removed. The apparently large long-term pain effect rests on one high-risk trial whose sample did not meet the optimal information size, and it describes a single appliance rather than conservative care in general. And for jaw function and oral-health-related quality of life the correct reading is an absence of usable evidence rather than evidence of absence: the intervals are wide enough to contain important benefit and important harm simultaneously.

## 4. Discussion

### 4.1. Summary of Main Findings

The review found a modest average short-term reduction in pain across conservative and minimally invasive interventions, but with very-low certainty and substantial between-study variability. The pooled difference of 1.31 points on a 0–10 scale was close to the operational threshold and fell to 0.94 points when high-risk results were removed. Short-term mouth opening increased by 1.96 mm and fell to 0.75 mm after high-risk results were excluded; the latter confidence interval included no difference. Evidence for jaw function, oral-health-related quality of life and sustained effects was sparse. The single most informative statistic is not any of the point estimates but the dispersion around them: the standard deviation of true effects across studies was 1.50 points for pain and 3.51 mm for opening, equal to or larger than the reference values used for interpretation. An average drawn from such dispersion describes the literature, not a patient.

The study-level intervention table makes the clinical basis of this heterogeneity explicit. Trials differed not only by treatment label, but by exposure: one manipulation, one TENS session, seven daily PBM sessions, 12 laser sessions, 8 weeks of supervised and home exercise, or continuous splint wear for up to 8 months. Injectable studies ranged from saline-controlled PRP or dry needling to botulinum toxin regimens of 100–150 U. A single overall estimate therefore summarizes a broad treatment question rather than a common biological dose.

### 4.2. Comparison with Previous Reviews

Table 6 compares the present estimates with previous syntheses. Direct numerical comparison requires caution because the reviews differ in diagnostic criteria, comparator type, outcome definition and whether they analyze change scores or final between-group values.

A review with a broadly similar scope reported favorable short-term effects across 10 studies [95], but it included nonrandomized designs, studies without RDC/TMD or DC/TMD diagnoses and active comparators. It therefore addressed a broader clinical question than the inactive-control analyses reported here.

With criteria-based diagnoses, inactive controls and result-specific risk-of-bias assessment, the present review reached a more cautious interpretation: the mean pain benefit was 1.31 points, but the prediction interval included no benefit.

**Figure 7:**
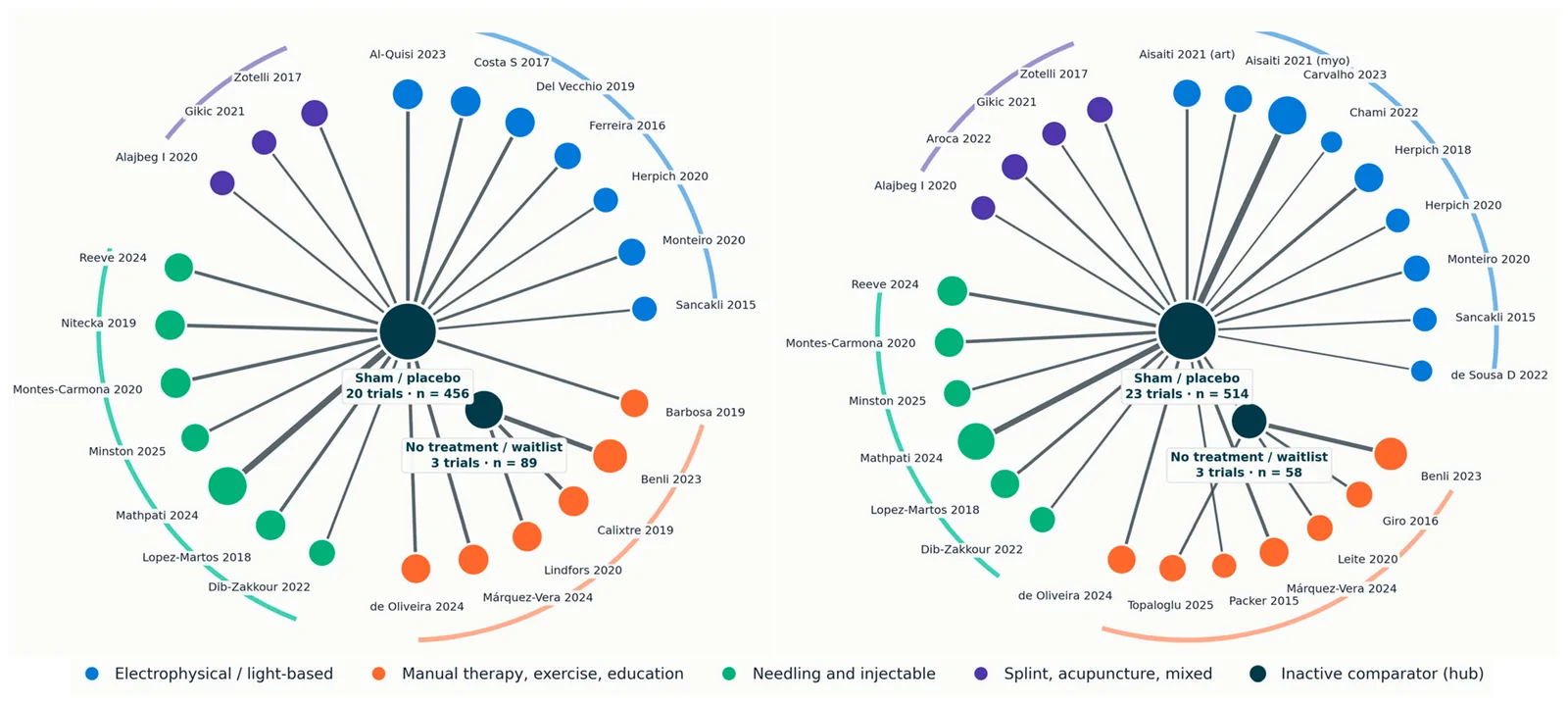
Structure of the comparisons for the two principal analyses: (**left**) short-term pain, 23 trials; (**right**) short-term mouth opening, 26 effects from 25 trials. Node area is proportional to the analyzed sample and edge width to the participants contributing that contrast. No edge joins two active treatments, because the review admits only inactive comparators.

Figure 7 shows why this review cannot rank treatments. Network meta-analysis requires direct or indirect connections between active treatments. Here, every eligible contrast compares a treatment with an inactive control; no network meta-analysis was undertaken.

Most short-term comparisons used sham or placebo controls rather than no treatment, with the aim of controlling contextual and placebo effects. The broad pooled estimates do not establish a comparative hierarchy.

Previous reviews of manual therapy, exercise, education and psychological care generally report possible benefits but low or very-low certainty and mixed comparators [15,16,17,21,22,94,96,97,98,99]. These questions differ from the present final-value comparisons against inactive controls.

Reviews of photobiomodulation, electrical stimulation and splints likewise emphasize protocol heterogeneity and uncertain effects [14,18,23,24,100,101,102]. The present results do not support ranking these options.

Network and intervention-specific syntheses of injections, acupuncture and other treatments may estimate relative effects across active comparators [11,12,13,19,25,26,92,93]. Those rankings cannot be transferred directly to this inactive-control estimand.

The broadly scoped review by Iturbe Cordero et al. included quasi-randomized designs and wider diagnostic and comparator criteria [95]. Together with current guidance [9], these design differences help explain why the present conclusions are more cautious.

### 4.3. Clinical Interpretation

Statistical significance does not establish clinical importance. TMD thresholds vary by outcome definition, instrument and follow-up, so they were used here as contextual benchmarks rather than universal cutoffs [47,48,49,53].

Published estimates include 1.95 points and a 37.9% relative reduction for chronic TMD pain [53], approximately 0.9–1.9 points for pain and 2.5 mm for mouth opening [47], 0.80 points for the pain MCID and 2.18 points for substantial clinical benefit [48], and 3.5 mm for pain-free mouth opening in muscular TMD [49].

Against pain benchmarks of 0.8, 1.0 and 1.95 points, the main estimate of 1.31 points exceeded the two lower values but not 1.95, whereas the lower-bias estimate of 0.94 points exceeded only 0.8. Both the main mouth-opening estimate of 1.96 mm and the lower-bias estimate of 0.75 mm were below 2.5- and 3.5 mm benchmarks. These values were developed largely as patient-level change thresholds or measurement benchmarks, not validated between-group treatment thresholds. The sensitivity pattern therefore supports cautious interpretation rather than a binary claim of clinically important or trivial benefit.

### 4.4. Intervention Families in Detail

#### 4.4.1. Broad Conservative Care and Occlusal Splints

The direction of the present pain estimate is consistent with the network meta-analysis by Yamaguchi et al., which included 24 trials for pain and found favorable estimates for low-level laser therapy, self-exercise and stabilization splints [20]. That analysis nevertheless rated the certainty as very low, and its mouth-opening estimates were imprecise. The present review reaches a similar certainty judgment while adding inactive-comparator restriction, outcome-specific RoB 2 assessment, prediction intervals and sensitivity analyses that remove high-risk results.

For occlusal interventions specifically, the 2024 Cochrane review included 57 studies and 2846 participants but identified only one study at low risk of bias and judged all principal comparisons very-low certainty [24]. Its inconclusive findings align with the present inability to infer a treatment-class ranking. The two splint-only studies in the current review used materially different appliances and exposures—a nocturnal stabilization splint for 6 months and complementary day/night repositioning devices for 8 months—while the multi-arm trials used thin foil or sham acupuncture as controls. Treating these regimens as one uniform intervention would conceal clinically relevant differences.

#### 4.4.2. Exercise and Manual Therapy

Idáñez-Robles et al. reported improvements in pain and active mouth opening across 16 exercise studies [22], while Villar-Aragón-Berzosa et al. found differences in disability and opening, but not pain or quality of life, when manual therapy was compared with splints [21]. These findings are not directly interchangeable with ours because the first review included add-on comparisons and the second compared two active treatments; the present review required an inactive control.

The ten manual/exercise/education studies in the present corpus were also clinically dissimilar. They included jaw resistance training, cervical motor control, cervical mobilization, massage, thoracic manipulation, diacutaneous fibrolysis, self-care education and whole-chain myofascial release. Treatment exposure ranged from a single session to daily home exercise. This diversity supports the broad pooled question but limits any inference that the pooled effect belongs to a particular technique.

#### 4.4.3. Photobiomodulation and Other Physical Agents

Photobiomodulation was the most frequent intervention family, yet its protocols were among the most variable. Wavelengths ranged from 635 to 905 nm; studies used laser, LED or combined sources; energy densities and application sites differed; and schedules ranged from one application to 14 sessions. The parameter-focused review by de Oliveira-Souza et al. included 89 studies, found 80.9% at high risk of bias and reported low- or moderate-certainty effects that varied with device and dose [23]. The present table shows that even trials placed in the same broad PBM category often do not represent the same treatment exposure. This is a plausible clinical source of heterogeneity and explains why a broad modality label is insufficient for dose–response interpretation.

#### 4.4.4. Needling, Injectable Treatments and Acupuncture

A 2024 meta-analysis of 15 botulinum toxin trials reported pain reductions of approximately 1.9 points at 1 month and 2.1 points at 6 months versus placebo [25]. The three botulinum toxin studies in the present review illustrate why results can differ: two 100-U saline-controlled trials found little between-group separation, whereas a smaller study pooling botulinum toxin and lidocaine against saline reported larger effects. Differences in case definition, injection sites, coexisting arthralgia, follow-up and analytical choices may all contribute. The current broad meta-analysis was not designed to resolve an optimal toxin dose or to combine botulinum toxin separately from other interventions.

The 2026 acupuncture meta-analysis by Ning et al. reported standardized improvements in pain and mouth opening across 19 trials [26]. It pooled change scores across different control types and included both needle and laser acupuncture. The current review contained three acupuncture studies with nonpenetrating or irrelevant-point controls and different schedules, and they contributed to different outcome syntheses. The two reviews are therefore complementary: the intervention-specific analysis estimates an acupuncture-class effect under broader assumptions, whereas the present review locates acupuncture within a consistent inactive-comparator framework without claiming a class-specific estimate.

#### 4.4.5. Interventions Underrepresented in This Review

Cognitive behavioral therapy and structured psychosocial care, which received strong recommendations in the linked guideline, do not appear in this review, while education or explicit home self-management components appear in only two eligible trials [69,75]. This pattern reflects both the eligibility criteria and the difficulty of constructing a comparator that is credible, attention-matched and therapeutically inert for behavioral interventions. Their limited representation must not be read as evidence against them. Readers should consult dedicated syntheses [97,98,99] and the guideline [9].

### 4.5. Clinical Sources of Variation Between Studies

Four clinical variables account for an identifiable share of the dispersion.

The first is phenotype. Short-term pain effects were more than twice the operational reference value in trials restricted to myogenous TMD (2.16 points) and indistinguishable from zero in mixed-phenotype trials (0.33 points), a difference of 1.84 points (*p* = 0.004 with modified Hartung–Knapp inference). Heterogeneity nevertheless remained substantial within both strata when fitted separately (τ = 1.26 points in myogenous trials and τ = 1.09 points in mixed-phenotype trials), so phenotype shifted the location of the effect without homogenizing it. This is an ecological, study-level signal, sensitive to the classification rule, and it is offered as a hypothesis for stratified trials rather than as a treatment-selection rule.

The second is the follow-up window. A window defined as ‘up to three months’ placed a measurement taken ten minutes after a single needling session in the same analysis as eight weeks of supervised exercise and three months of nocturnal splint wear. This is not a statistical subtlety: an immediate post-procedural change and a treatment course are different clinical phenomena with different mechanisms and different relevance to a patient. Restricting the window reduced the between-study standard deviation (τ) from 3.51 to 1.97 mm (a 44% decrease) and I^2^ by 7.6 percentage points, from 85.9% to 78.3%, although substantial heterogeneity remained; it also reduced the width of the prediction interval to approximately 58% of its original width. This indicates that part of the apparent variability reflects measurement timing—an identifiable and correctable design feature.

The third source of heterogeneity is comparator content. Saline injection, a switched-off laser, nonpenetrating acupuncture and a thin foil device control for procedural context to different degrees, whereas waitlist or no treatment does not. A device-versus-no-device trial measures the active component together with differences in expectation and attention that are controlled more effectively in an otherwise identical sham-controlled trial.

The fourth factor is outcome definition. Pain-free, maximum unassisted and maximum assisted mouth opening are related but not interchangeable. Because assistance and stopping criteria were often incompletely reported, the available study-level data did not support separate analyses for each construct.

A fifth factor could not be quantified here but probably matters as much as any of the above: baseline severity. Several trials enrolled participants whose mouth opening was already within the normal range, in which a 3.5 mm between-group gain is neither achievable nor clinically meaningful. In one trial the authors themselves attributed their null range-of-motion finding to the fact that only three of eighteen participants had any opening limitation before treatment, with baseline values of 42.8 to 44.0 mm [71]. Pooled opening estimates in this literature are therefore constrained in part by a ceiling effect, and should not be read as evidence that these interventions cannot improve opening in patients who are actually restricted. Baseline severity was not reported consistently enough across the corpus to support a meta-regression, which is itself a reporting failure worth naming.

### 4.6. Trustworthiness of the Evidence

Risk of bias further widened uncertainty. None of the 73 result-specific assessments was low risk overall, and excluding high-risk results reduced the pooled effects, especially for mouth opening. Selective-reporting concerns, missing outcome-specific denominators and insufficiently specified measurement procedures were not evenly distributed across studies, so they can contribute to both effect inflation and heterogeneity. The registry discrepancies documented in the Results are not isolated curiosities: they indicate that prespecification in this field is frequently nominal. A registration number in an abstract was, in the cases we checked directly, a weak guarantee that a prospective analysis plan existed, that the reported primary outcome was the registered one, or that the participant flow in the figure matched the participant flow in the analysis. Comparable proportions have been reported elsewhere—80.9% of 89 photobiomodulation trials at high risk of bias [23], one low-risk study among 57 occlusal-intervention trials [24], and no low-risk trial at all among 14 trials of myofascial pain interventions [93]—which suggests a field-wide problem rather than a peculiarity of our sample.

### 4.7. Implications for Rehabilitation Practice and Translational Research

Three statements follow from this synthesis and can be offered to multidisciplinary rehabilitation teams without overreaching. First, averaged across device- and procedure-based conservative treatments, the benefit over an inactive control is small for pain and, for mouth opening, not distinguishable from no effect after high-risk results are excluded. Second, the dispersion between studies is as large as the effect sought, so the average should not be used as an expected value for an individual patient; the prediction intervals, which include no benefit for every principal analysis, are the more honest summary to carry into a consultation. Third, the interventions that current guidance recommends most strongly—education, reassurance, self-management, supervised exercise and cognitive behavioral therapy—are underrepresented here because they are difficult to test against a credible sham, not because they failed. The practical emphasis should remain on shared functional goals and first-line reversible care, while the uncertain incremental benefit of a device or injection is discussed openly. The study-level myogenous–mixed interaction is exploratory and should not be used to select treatment for an individual patient.

Failure to demonstrate a clear incremental benefit over inactive controls does not show that conservative care is ineffective or should be withheld.

### 4.8. Strengths and Limitations

The main strengths are the verification of diagnoses, outcomes, treatment exposure and comparators against primary reports; independent RoB 2 and GRADE assessment by two human reviewers; appropriate handling of shared controls; and sensitivity and prediction analyses. Reporting τ alongside I^2^ also shows whether differences among study results are large enough to matter clinically.

The main limitation is the breadth and quality of the evidence base. The pooled estimate combines treatments with different mechanisms and doses and therefore cannot establish the comparative efficacy of individual interventions. Several categories contained too few similar trials for stable subgroup estimates, and two analyses contained only two studies. Baseline severity and follow-up beyond three months were reported inconsistently, while function and OHRQoL were less complete than pain or opening. Some effects required conversion from medians or extraction from figures. Change-score or ANCOVA sensitivity analyses were not possible because the required variances, correlations or adjusted estimates were unavailable. Mouth-opening definitions were also incompletely reported, and aggregate data cannot identify individual responders or fully separate treatment effects from differences in baseline severity and co-interventions.

The review was limited to English-language reports published in 2015–2025. The lower date boundary follows the 2014 publication of DC/TMD, while RDC/TMD studies remained eligible; nevertheless, the date and language restrictions may introduce selection and language bias. Later reviews were used only for context. Treatment details in Table 2 were checked against the primary reports, and missing information was not inferred.

### 4.9. Research Priorities

The evidence gaps concern both study design and clinical reporting. Prospective protocols, concealed allocation, outcome-specific participant flow and complete numerical results would reduce avoidable bias. Intervention reports should specify the treatment site, dose, session frequency, total exposure, home adherence and background care. Pain-free, maximum unassisted and assisted opening should be reported separately, together with baseline severity. Follow-up should reflect treatment exposure so that immediate procedural responses are not pooled with completed treatment courses. Function, OHRQoL and follow-up beyond six months remain underrepresented; core-outcome-set work may improve consistency [103]. Adequately powered trials are also needed to test whether myogenous, arthrogenous and mixed phenotypes truly modify treatment response. Pragmatic studies across dental and rehabilitation services should examine adherence, access and participation-related outcomes so that efficacy can be translated into routine care.

## 5. Conclusions

Across 35 randomized studies, conservative and minimally invasive treatments produced a modest average reduction in short-term pain, and the estimate became smaller after high-risk results were removed. The mean improvement in mouth opening became inconclusive after high-risk results were excluded. Results varied as much as the average effects themselves, so the pooled estimates describe the literature rather than the result expected for an individual patient. Differences in phenotype, treatment duration, comparator and outcome definition help explain this variation. The review characterizes evidence against inactive controls; it does not establish comparative efficacy or a treatment hierarchy. For rehabilitation practice, the findings support patient-centered functional goals and transparent discussion of the uncertain incremental benefit of device- or procedure-based options within first-line reversible care.

## Figures and Tables

**Table 1:** Clinical eligibility and outcome framework.

Element	Eligibility Used in This Review
Population	Adults with TMD diagnosed using DC/TMD or RDC/TMD
Intervention	Conservative or minimally invasive treatment
Comparator	Sham/placebo, no treatment or waitlist
Outcomes	Pain, mouth opening, jaw functional limitation and OHRQoL
Timing	Short ≤ 3 months; medium > 3–6 months; long > 6 months
Design	English-language, full-text, peer-reviewed randomized controlled trial report; 2015–2025

**Table 2:** Characteristics of the 35 studies included in the quantitative synthesis. n is the analyzed sample for eligible outcomes; ranges indicate outcome- or time-specific denominators or inconsistent reporting, not additional cohorts. In multi-arm trials, eligible active groups were combined against the shared inactive control for the overall analysis, while intervention details are shown separately. Study labels use the first online-publication year where applicable; the reference list follows the final issue year. PBM, photobiomodulation; TENS, transcutaneous electrical nerve stimulation; BTX-A, botulinum toxin type A; PRP, platelet-rich plasma; JFL, jaw functional limitation; OHRQoL, oral-health-related quality of life.

Study	Diagnosis/Phenotype	n	Intervention and Exposure	Comparator	Contribution to Synthesis
**Aisaiti 2021 [57]**	DC/TMD; separate myogenous and arthrogenous cohorts	100	PBM (GaAlAs 810 nm, 6 J/cm^2^): masseter, 3 regions ×20 s; TMJ, 5 points ×6 s; once daily for 7 days	Device-off placebo	Opening; day 7
**Al-Quisi 2023 [58]**	DC/TMD; myogenous	60	Red LED (660 nm, 1.6 W, 5 min/tender site) or diode laser (810 nm, 1 W, 30 s/affected point); each given at 3 weekly visits	Device-off light/laser	Pain; short term
**Alajbeg 2020 [59]**	DC/TMD; mixed	30	Full-coverage hard-acrylic maxillary stabilization splint, worn nightly for 6 months (hours/night not reported)	0.5 mm thin placebo splint	Pain and opening; 3 and 6 months
**Aroca 2022 [60]**	RDC/TMD; myogenous	41	Auricular acupuncture (TMJ point 43.3; maximum 48 h retention; 8 weekly applications) or occlusal splint (8 h/night; total duration not reported)	Sham ear acupuncture at an irrelevant point	Opening; 8 weeks
**Barbosa 2019 [61]**	RDC/TMD; myogenous	46	Progressive masticatory resistance/endurance exercise with biofeedback (20–50% MVIC); twice weekly for 8 weeks (16 sessions)	Simulated laser	Pain; 8 weeks
**Benli 2023 [62]**	DC/TMD; myogenous	90	Lavender-oil or almond-oil standardized massage (6 mL and approximately 50 min/session); twice weekly for 8 weeks (15 sessions)	No massage/no treatment	Pain and opening; post-treatment/2 months
**Calixtre 2019 [63]**	RDC/TMD; mixed	61	Upper-cervical mobilization plus pressure-biofeedback craniocervical flexor training (initial 20–22 mmHg); 10 sessions over 5 weeks	No treatment	Pain and JFL; 5 weeks
**Carvalho 2023 [64]**	RDC/TMD; phenotype not reported	145	PBM (808 nm, 100 mW, 30 s, 3 J/point); twice weekly for 4 weeks (8 sessions); synthesis used the first-session estimate	Sham PBM	Opening; immediate/short term
**de Oliveira Chami 2022 [65]**	RDC/TMD; myogenous	18	GaAlAs LLLT (808 nm, 100 mW, 80 J/cm^2^, 22 s and 2.2 J/painful point); 2 sessions 48 h apart	Sham LLLT	Opening and OHRQoL; 30 days
**Costa 2017 [66]**	RDC/TMD; mixed	60	Single PBM session (830 nm, 100 mW, 100 J/cm^2^, 28 s and 2.8 J/point) to masticatory-muscle sites	Sham PBM	Pain; immediate/short term
**D’Attilio 2021 [67]**	DC/TMD; myogenous	86	Personalized mandibular repositioning: MORA during the day except meals and SVED all night; 8 months	No treatment	Pain; 8 months
**Del Vecchio 2019 [68]**	RDC/TMD; phenotype not reported	57	Home LLLT (808 nm, 250 mW, 40 J/application over the painful area for 8 min); twice daily for 7 days (14 sessions; 560 J/course)	Identical sham device	Pain; 1 week
**de Oliveira-Souza 2024 [69]**	RDC/TMD; mixed	54	Neck motor-control training (30 min weekly supervision plus 15–20 min daily home exercise) or manual therapy (30–40 min weekly plus home self-care); 8 weeks	Switched-off therapeutic ultrasound	All four outcomes; post-treatment to 3-month follow-up
**de Salles-Neto 2020 [70]**	RDC/TMD; myogenous	32	Standardized acupuncture (16 insertions retained 20 min); once weekly for 5 weeks	Nonpenetrating sham acupuncture	JFL and OHRQoL; 1 month
**de Sousa 2022 [71]**	RDC/TMD; mixed	18	Combined red/infrared LED (660/850 nm): 75.6 J per wavelength array, 151.2 J/session combined, 1200 s; 3 sessions/week for 2 weeks (907.2 J/course)	Device-off LED	Opening; after 6 sessions
**Dib-Zakkour 2022 [72]**	RDC/TMD-derived criteria; mixed	36	One bilateral deep dry-needling session of masseter trigger points (0.30 × 30 mm needle; insertion count not reported)	Simulated needling without penetration	Pain and opening; 10 min
**Ferreira 2016 [73]**	RDC/TMD; mixed	40	One 50 min active TENS session to both masseters (4 Hz for 25 min, then 100 Hz for 25 min)	Placebo TENS with current faded after 40 s	Pain; immediate and 48 h
**Gikić 2021 [74]**	DC/TMD; mixed	30	Home physical therapy (10–20 repetitions/exercise twice daily) or nocturnal hard-acrylic stabilization splint; 6 months	0.5 mm nocturnal thermoformed foil	Pain/opening at 3/6 months; JFL/OHRQoL at 6 months
**Giro 2016 [75]**	RDC/TMD; mixed	42	Education alone (standardized video/print) or education plus self-care (15 min moist heat, 6-repetition exercises and self-massage 3 times/day); 30 days	No treatment before the analyzed time point	Opening; 30 days
**Herpich 2018 [76]**	RDC/TMD; phenotype not reported	60	Single combined phototherapy session (905 nm laser plus 640/875 nm LEDs) at 2.62, 5.24 or 7.86 J/point; 10 points total	Sham phototherapy	Opening; up to 48 h
**Herpich 2020 [77]**	RDC/TMD; myogenous	30	Intraoral combined phototherapy (905 nm laser plus 640/875 nm LEDs), 39.27 J/side over 300 s; 6 sessions over 2 weeks	Sham phototherapy	Pain and opening; after 6 sessions
**Leite 2020 [78]**	RDC/TMD; myogenous	40	Diacutaneous fibrolysis of masseter/temporalis using a hook; approximately 10 min, twice weekly for 4 weeks (8 sessions)	Superficial sham fibrolysis	Opening; 4 weeks
**Lindfors 2020 [79]**	RDC/TMD; mixed (myofascial pain; concurrent joint diagnoses permitted)	56	Relaxation, free jaw movement, light resistance and stretching exercises performed three times daily for 3 months (repetitions not reported)	Waitlist/no treatment	Pain; 3 months
**Lopez-Martos 2018 [80]**	RDC/TMD; myogenous	57	Lateral-pterygoid percutaneous needle electrolysis (2 mA ×3 s ×3 impacts) or deep dry needling; once weekly for 3 weeks	Sham pressure without penetration	Pain and opening; day 70
**Márquez-Vera 2024 [81]**	DC/TMD; mixed	61	Single mandibular muscle-energy session: 3 sets ×3 isometric closing contractions ×3 s, with passive opening between sets	Simulated suboccipital inhibition	Pain and opening; immediate
**Mathpati 2024 [82]**	DC/TMD (reference-linked); phenotype not reported	128	Single intra-articular PRP injection to the affected TMJ (injected volume/concentration not sufficiently reported)	Simulated placebo injection	Pain and opening; 8 weeks
**Minston 2025 [83]**	DC/TMD; mixed	45	One BTX-A session: 100 U across 14 bilateral masseter/temporalis sites	Volume-matched saline injections	Pain and opening; approximately 2 months
**Monteiro 2020 [84]**	RDC/TMD; myogenous	42	PBM (635 nm, 8 J/cm^2^, 200 mW, 20 s = 4 J/point/session) at painful points; 4 weekly sessions (16 J/point/course)	Identical protocol without laser activation	Pain and opening; 1 month
**Montes-Carmona 2021 [85]**	DC/TMD; myogenous	59–60	One EMG-guided session of BTX-A (100–150 U) or 2% lidocaine (total volume variable/not reported) into involved masseter, temporalis and pterygoid muscles	Saline injections	Pain/opening; days 90 and 180
**Nitecka-Buchta 2019 [86]**	RDC/TMD; myogenous	58	Single bilateral intramuscular PRP injection at three painful masseter points/side (0.5 mL/point; 3 mL total)	Volume-matched 0.9% saline	Pain; day 14
**Packer 2015 [87]**	RDC/TMD; mixed	32	Single upper-thoracic high-velocity manipulation at T1; one repeat only if no cavitation (maximum 2 attempts)	Position-matched sham manipulation without thrust/cavitation	Opening; immediate and 2–4 days
**Reeve 2024 [88]**	DC/TMD; myogenous	50–66	One onabotulinumtoxinA session (100 U: 37.5 U/masseter and 12.5 U/temporalis bilaterally)	Volume-matched saline injections	Pain/opening/JFL; 1–3 months
**Sancakli 2015 [89]**	RDC/TMD; myogenous	30	LLLT (820 nm, 3 J/cm^2^, 300 mW, 10 s/point) at greatest-pain or predetermined muscle sites; 12 sessions over 1 month	Device-on but nonemitting placebo	Pain and opening; post-treatment
**Şenel Topaloğlu 2025 [90]**	DC/TMD (supported by cited diagnostic protocol); phenotype not reported	45	Myofascial release (10 sessions) or structured exercise; 4 weeks (session duration/repetition dose not fully reported)	No treatment	Opening; 4 weeks
**Zotelli 2017 [91]**	RDC/TMD; mixed	40	Real needle acupuncture (0.30 × 30 mm) at ST6, ST7, SI18, GV20, GB20, BL10 and LI4; 20 min once weekly for 4 weeks	Nonpenetrating sham acupuncture	Pain and opening; post-treatment

**Table 3:** Between-study variation in eight of the nine analyses. Long-term pain included one study, so heterogeneity could not be estimated. For analyses with only two studies, heterogeneity metrics are shown as dashes because they are not reliably estimable. For analyses with three or four studies, estimates are shown but remain unstable; a value of zero does not establish homogeneity. τ is the estimated standard deviation of true effects on the outcome scale and can be compared with the operational clinical threshold. MD, mean difference; g, standardized effect.

Analysis	k	Metric	τ^2^	τ (Outcome Scale)	I^2^	Operational Reference
**Pain, short term**	23	MD	2.25	1.50 points	93.6%	1 point
**Pain, medium term**	4	MD	0.00	0.00 points	0%	1 point
**Opening, short term**	26	MD	12.32	3.51 mm	85.9%	3.5 mm
**Opening, medium term**	4	MD	0.66	0.81 mm	14.2%	3.5 mm
**Jaw function, short term**	4	g	0.055	0.23	36.6%	0.20
**Jaw function, medium term**	2	g	—	—	—	0.20
**OHRQoL, short term**	3	g	0.00	0.00	0%	0.20
**OHRQoL, medium term**	2	MD	—	—	—	5 OHIP-14 points

**Table 4:** Summary of the nine outcome-by-follow-up analyses and final GRADE certainty. Effects are worded in the direction of benefit. OHRQoL, oral-health-related quality of life; CI, confidence interval; g, standardized effect.

Outcome/Timing	Studies/Participants	Average Effect (95% CI)	Certainty (GRADE)	Clinical Interpretation
Pain, short term	23/1221	1.31 points better (0.62 to 2.00)	Very low	Possible modest benefit; highly variable
Pain, medium term	4/173	1.12 points better (0.23 to 2.02)	Low	Possible modest benefit; few studies
Pain, long term	1/86	4.35 points better (3.53 to 5.17)	Very low	Single high-risk study
Opening, short term	26 effects/25 studies; 1340	1.96 mm greater (0.33 to 3.60)	Very low	Inconclusive after excluding high-risk results
Opening, medium term	4/173	2.25 mm greater (−1.60 to 6.10)	Very low	No clear difference
Jaw function, short term	4/197	g 0.31 better (−0.29 to 0.91)	Very low	No clear difference
Jaw function, medium term	2/84	g 0.24 better (−2.71 to 3.20)	Very low	Very uncertain
OHRQoL, short term	3/104	g 0.17 better (−0.70 to 1.04)	Very low	No clear difference
OHRQoL, medium term	2/84	2.29 OHIP-14 points better (−31.30 to 35.89)	Very low	Very uncertain

**Table 5:** Summary of findings for seven clinically prioritized efficacy outcomes. Effects are worded in the direction of benefit. Complete GRADE explanations are provided in Supplementary Table S2. The two medium-term jaw function and OHRQoL analyses remain in Table 4. CI, confidence interval; g, Hedges standardized effect; OHRQoL, oral-health-related quality of life.

Outcome/ Follow-Up	Studies/Participants	Effect (95% CI)	GRADE	Main Reasons for Downgrading
Pain—short term (≤3 months)	23/1221	1.31 points better (0.62 to 2.00)	Very low	Risk of bias (10 of 23 results high, none low; estimate falls to 0.94 points without them); inconsistency (I^2^ 93.6%, τ 1.50 points exceeds the 1-point threshold, prediction interval spans benefit and harm); imprecision (CI crosses the 1-point threshold).
Pain—medium term (>3 to ≤6 months)	4/173	1.12 points better (0.23 to 2.02)	Low	Risk of bias (1 of 4 results high, none low; removing it makes the CI include no effect); imprecision (CI crosses the 1-point threshold).
Pain—long term (>6 months)	1/86	4.35 points better (3.53 to 5.17)	Very low	Risk of bias (a single high-risk trial, no replication); imprecision (optimal information size not met: about 117 participants would be needed at 80% power and 157 at 90% for a 1-point difference with pooled SD 1.93). Applies to one personalized repositioning appliance, not to conservative care generally.
Mouth opening—short term (≤3 months)	26 effects/25 studies; 1340	1.96 mm greater (0.33 to 3.60)	Very low	Risk of bias (9 of 26 results high, none low; 0.75 mm without them, CI then including zero); inconsistency (I^2^ 85.9%, τ 3.51 mm equals the 3.5 mm threshold); indirectness (pain-free, unassisted and assisted opening pooled); imprecision (CI crosses the 3.5 mm threshold).
Mouth opening—medium term (>3 to ≤6 months)	4/173	2.25 mm greater (−1.60 to 6.10)	Very low	Risk of bias (all four results with some concerns, none low); indirectness (opening constructs and stopping criteria not uniform); imprecision (CI crosses both the 3.5 mm benefit boundary and no effect).
Jaw functional limitation—short term (≤3 months)	4/197	g 0.31 better (−0.29 to 0.91)	Very low	Risk of bias (2 of 4 results high, none low); inconsistency (τ 0.23 standardized units exceeds the 0.20 threshold); very serious imprecision (CI includes important benefit and important harm; 197 participants).
OHRQoL—short term (≤3 months)	3/104	g 0.17 better (−0.70 to 1.04)	Very low	Risk of bias (all three results with some concerns, none low); very serious imprecision (CI includes important benefit and important harm; 104 participants).

**Table 6:** The present estimates in the context of previous syntheses. Clinical questions differ: this review includes only inactive comparators and analyzes final between-group values. NMA, network meta-analysis; SR, systematic review; MA, meta-analysis; RoB, risk of bias; SUCRA, surface under the cumulative ranking curve.

Synthesis	Scope and Clinical Question	Trials/Participants	Reported Effect	Risk of Bias/Certainty
**Yao et al. 2023 [19]; guideline [9]**	All interventions for chronic TMD pain; network of active and inactive comparators	153/8713; 59 interventions	Guideline recommends for CBT, mobilization, trigger-point therapy, supervised exercise, usual care; conditionally against LLLT, TENS, botulinum toxin, reversible splints, trigger-point injection	Certainty varies by node; recommendations mostly conditional
**Yamaguchi et al. 2025 [20]**	Initial conservative care; NMA	24 trials for pain	Favorable pain estimates for LLLT, self-exercise, stabilization splints; opening estimates imprecise	Very low certainty
**Singh et al. 2024 (Cochrane) [24]**	Occlusal interventions; SR/MA	57/2846	Inconclusive for all principal comparisons	One low-RoB study; very low certainty
**de Oliveira-Souza et al. 2023 [23]**	Low-level laser parameters; SR/MA	89 trials	Effects vary with device and dose	80.9% at high RoB; low to moderate certainty
**Li et al. 2024 [25]**	Botulinum toxin versus placebo; SR/MA	15 trials	Approximately 1.9 points at 1 month and 2.1 points at 6 months	Not stated here
**Ning et al. 2026 [26]**	Acupuncture; SR/MA of change scores, mixed control types	19 trials	Standardized improvements in pain and mouth opening	Not stated here
**Idáñez-Robles et al. 2023 [22]**	Exercise therapy; SR/MA, includes add-on comparisons	16/812	Standardized improvements in pain and active mouth opening	Not stated here
**Villar-Aragón-Berzosa et al. 2024 [21]**	Manual therapy versus occlusal splint; active versus active	9 trials	Differences for disability and opening; none for pain or quality of life	Not stated here
**Menéndez-Torre et al. 2023 [92]**	Deep dry needling versus manual therapy; NMA	8/556	No difference between the two; manual therapy better than placebo (−1.32, 95% CI −2.19 to −0.46) and than botulinum toxin (−1.72, 95% CI −3.08 to −0.36)	Mean PEDro 6.71/10; I2 65.3%; low quality
**Ma et al. 2026 [93]**	Myofascial TMD pain; Bayesian NMA with ranking	14/674	SUCRA: lidocaine injection 91.7%, ischaemic compression 88.7%, PRP 68.6%, electrical stimulation 67.5%	64.3% high RoB, no low-RoB trial; low to very low certainty
**González-Sánchez et al. 2020 [94]**	Manual therapy; SR/MA, largely within-group change, no inactive-comparator restriction	6/304	Approximately 4 points for pain and approximately 15 mm for maximum mouth opening	No therapist blinding in any trial
Iturbe Cordero et al. 2025 [95]	Conservative and minimally invasive treatments; active comparators and standard care pooled together; randomized and non-randomized designs	10/approx. 500	Direction favoring active treatment for short-term pain and opening; no pooled estimate, confidence interval or I^2^ reported in the text	Study-level Cochrane risk of bias; GRADE not applied
**Present review**	Conservative and minimally invasive treatments; inactive comparator only, final values, outcome-specific RoB	35/69 effects/1221 (pain) and 1340 (opening)	Pain 1.31 points (0.62 to 2.00), 0.94 after removing high-RoB results; opening 1.96 mm (0.33 to 3.60), 0.75 mm after removing high-RoB results	No low-RoB result; 25 of 73 high RoB; very low certainty

## Data Availability

All data supporting the findings of this systematic review and meta-analysis are provided within the article and its Supplementary Materials. Additional study-level extraction data and analysis files underlying the reported results are available from the corresponding author upon reasonable request.

## Supplementary Materials

The following supporting information can be downloaded at: https://www.mdpi.com/article/10.3390/medsci14060660/s1, Table S1, complete database-specific search strategies; Figure S1A, RoB 2 summary; Figure S1B, RoB 2 traffic-light plots; Table S2, GRADE evidence profile; Table S3, individual-study results for the primary syntheses and exploratory treatment-cluster contrasts; Table S4, exploratory treatment-cluster contrasts; Data S1, analytic dataset; File S1, R code; File S2, completed PRISMA 2020 checklist. Trial registry records supporting the source documentation in Supplementary Table S3 are listed in References [104,105,106,107,108,109,110].

## Abbreviations

The following abbreviations are used in this manuscript:

ANCOVA	Analysis of covariance
BTX-A	Botulinum toxin type A
CBT	Cognitive behavioral therapy
CI	Confidence interval
DC/TMD	Diagnostic Criteria for Temporomandibular Disorders
GRADE	Grading of Recommendations Assessment, Development and Evaluation
JFL	Jaw functional limitation
JFLS-8	8-item Jaw Functional Limitation Scale
LED	Light-emitting diode
LLLT	Low-level laser therapy
MA	Meta-analysis
MCID	Minimal clinically important difference
MD	Mean difference
MORA	Mandibular orthopedic repositioning appliance
MVIC	Maximum voluntary isometric contraction
NMA	Network meta-analysis
OHIP-14	14-item Oral Health Impact Profile
OHRQoL	Oral-health-related quality of life
PBM	Photobiomodulation
PEDro	Physiotherapy Evidence Database scale
PICOS	Population, Intervention, Comparator, Outcomes, Study design
PRISMA	Preferred Reporting Items for Systematic Reviews and Meta-Analyses
PRISMA-P	Preferred Reporting Items for Systematic Review and Meta-Analysis Protocols
PROSPERO	International Prospective Register of Systematic Reviews
PRP	Platelet-rich plasma
RDC/TMD	Research Diagnostic Criteria for Temporomandibular Disorders
REML	Restricted maximum likelihood
RoB	Risk of bias
RoB 2	Cochrane Risk of Bias 2 tool
SR	Systematic review
SUCRA	Surface under the cumulative ranking curve
SVED	Sved appliance (anterior bite plane)
TENS	Transcutaneous electrical nerve stimulation
TMD	Temporomandibular disorders
TMJ	Temporomandibular joint
