# Supplementary material for: Conservative and Minimally Invasive Treatments for Temporomandibular Disorders Versus Inactive Controls: A Systematic Review and Meta-Analysis of Randomized Trials
Source: Med Sci (Basel). 2026 Oct 10;14(6):660. doi: 10.3390/medsci14060660 (PMC13706717; doi:10.3390/medsci14060660)
Supplement: Supplementary file 1 [file medsci-14-00660-s001.zip › Figure_S1A_RoB2_Summary.pdf]

Risk of bias 2 summary

Distribution of result-specific judgments (73 active assessments)

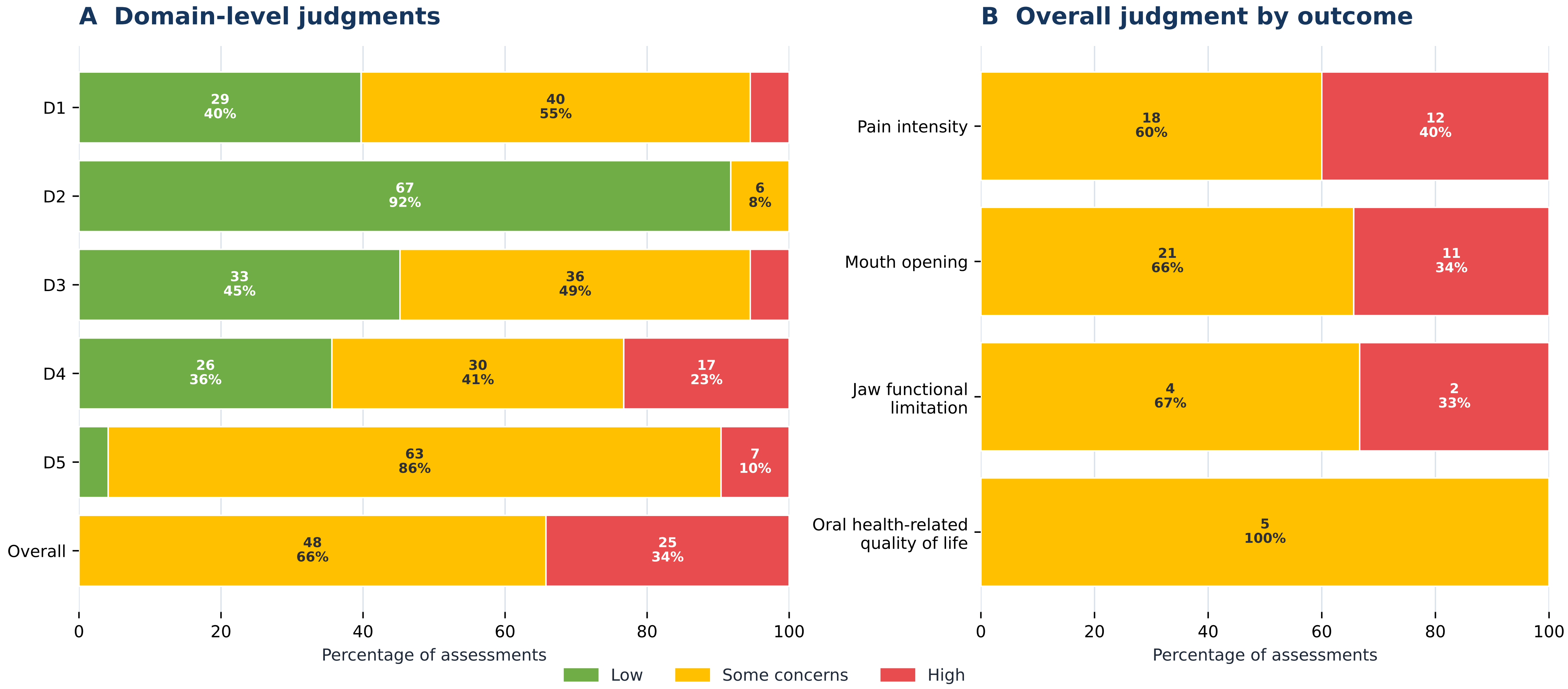

Percentages are calculated from the 73 active result-specific assessments; the retired parent record is excluded.
