# Supplementary material for: Conservative and Minimally Invasive Treatments for Temporomandibular Disorders Versus Inactive Controls: A Systematic Review and Meta-Analysis of Randomized Trials
Source: Med Sci (Basel). 2026 Oct 10;14(6):660. doi: 10.3390/medsci14060660 (PMC13706717; doi:10.3390/medsci14060660)
Supplement: Supplementary file 1 [file medsci-14-00660-s001.zip › Figure_S1B_RoB2_Traffic_Light.pdf]

RoB 2 traffic-light plot: Pain intensity

Result-specific assessments (n=30); effect of assignment to intervention

Study / result	D1 Randomization	D2 Deviations	D3 Missing data	D4 Measurement	D5 Reporting	Overall
Al-Quisi 2023 [PAIN-S-02] · Short	Some concerns	Low	Low	High	High	High
Alajbeg I 2020 [PAIN-S-01] · Short	Some concerns	Low	Some concerns	Low	Some concerns	Some concerns
Barbosa 2019 [PAIN-S-03] · Short	Low	Low	Some concerns	Some concerns	Some concerns	Some concerns
Benli 2023 [PAIN-S-04] · Short	Some concerns	Low	Low	High	Some concerns	High
Calixtre 2019 [PAIN-S-05] · Short	Low	Low	Low	High	Some concerns	High
Costa S 2017 [PAIN-S-06] · Short	Some concerns	Low	Low	Low	Some concerns	Some concerns
de Oliveira 2024 [PAIN-S-08] · Short	Low	Low	Some concerns	Some concerns	Some concerns	Some concerns
Del Vecchio 2019 [PAIN-S-07] · Short	Some concerns	Some concerns	Low	Low	Some concerns	Some concerns
Dib-Zakkour 2022 [PAIN-S-09] · Short	Some concerns	Low	Low	High	Some concerns	High
Ferreira 2016 [PAIN-S-10] · Short	Low	Low	Low	Some concerns	Some concerns	Some concerns
Gikic 2021 [PAIN-S-11] · Short	Some concerns	Low	Some concerns	Some concerns	Some concerns	Some concerns
Gikic 2021 [PAIN-S-11-PT] · Short	Some concerns	Low	Some concerns	Some concerns	Some concerns	Some concerns
Gikic 2021 [PAIN-S-11-SS] · Short	Some concerns	Low	Some concerns	Low	Some concerns	Some concerns
Herpich 2020 [PAIN-S-12] · Short	Low	Low	Low	Low	Some concerns	Some concerns
Lindfors 2020 [PAIN-S-13] · Short	High	Low	Some concerns	High	Some concerns	High
Lopez-Martos 2018 [PAIN-S-14] · Short	Some concerns	Low	Low	Some concerns	Some concerns	Some concerns
Mathpati 2024 [PAIN-S-16] · Short	Some concerns	Low	Some concerns	High	Some concerns	High
Minston 2025 [PAIN-S-17] · Short	Low	Some concerns	Low	Low	Some concerns	Some concerns
Monteiro 2020 [PAIN-S-18] · Short	Some concerns	Low	Low	High	Some concerns	High
Montes-Carmona 2020 [PAIN-S-19] · Short	Some concerns	Low	Low	High	Some concerns	High
Márquez-Vera 2024 [PAIN-S-15] · Short	High	Low	Low	High	High	High
Nitecka 2019 [PAIN-S-20] · Short	Some concerns	Low	Low	Some concerns	Some concerns	Some concerns
Reeve 2024 [PAIN-S-21] · Short	Low	Low	High	Low	Low	High
Sancakli 2015 [PAIN-S-22] · Short	Some concerns	Low	Some concerns	Low	Some concerns	Some concerns
Zotelli 2017 [PAIN-S-23] · Short	Some concerns	Low	Low	Some concerns	Some concerns	Some concerns
Alajbeg I 2020 [PAIN-M-01] · Medium	Some concerns	Low	Some concerns	Low	Some concerns	Some concerns
de Oliveira 2024 [PAIN-M-02] · Medium	Low	Low	Some concerns	Some concerns	Some concerns	Some concerns
Gikic 2021 [PAIN-M-03] · Medium	Some concerns	Low	Some concerns	Some concerns	Some concerns	Some concerns
Montes-Carmona 2020 [PAIN-M-04] · Medium	Some concerns	Low	Low	High	Some concerns	High
D'Attilio 2021 [PAIN-L-01] · Long	Some concerns	Low	Low	High	Some concerns	High

Low   Some concerns   High

D1 randomization; D2 deviations from intended interventions; D3 missing outcome data; D4 outcome measurement; D5 selection of the reported result.

RoB 2 traffic-light plot: Mouth opening

Result-specific assessments (n=32); effect of assignment to intervention

Study / result	D1 Randomization	D2 Deviations	D3 Missing data	D4 Measurement	D5 Reporting	Overall
Aisaiti 2021 [OPEN-S-02-ART] · Short	+	+	−	+	?	−
Aisaiti 2021 [OPEN-S-02-MYO] · Short	+	+	?	+	?	?
Alajbeg I 2020 [OPEN-S-03] · Short	?	+	?	+	?	?
Aroca 2022 [OPEN-S-04] · Short	?	+	?	?	−	−
Aroca 2022 [OPEN-S-04-AA] · Short	?	+	?	+	−	−
Aroca 2022 [OPEN-S-04-OS] · Short	?	+	?	?	−	−
Benli 2023 [OPEN-S-05] · Short	?	+	+	?	?	?
Carvalho 2023 [OPEN-S-06] · Short	+	+	+	+	?	?
Chami 2022 [OPEN-S-01] · Short	+	?	?	+	?	?
de Oliveira 2024 [OPEN-S-07] · Short	+	+	?	?	?	?
de Sousa D 2022 [OPEN-S-08] · Short	?	?	−	+	?	−
Dib-Zakkour 2022 [OPEN-S-09] · Short	?	+	+	?	?	?
Gikic 2021 [OPEN-S-10] · Short	?	+	?	?	?	?
Giro 2016 [OPEN-S-11] · Short	?	+	?	−	?	−
Herpich 2018 [OPEN-S-12] · Short	+	+	+	+	?	?
Herpich 2020 [OPEN-S-13] · Short	+	+	+	+	?	?
Leite 2020 [OPEN-S-14] · Short	+	+	+	?	−	−
Lopez-Martos 2018 [OPEN-S-15] · Short	?	+	+	?	?	?
Mathpati 2024 [OPEN-S-17] · Short	?	+	?	−	?	−
Minston 2025 [OPEN-S-26] · Short	+	?	+	+	?	?
Monteiro 2020 [OPEN-S-18] · Short	?	+	+	−	?	−
Montes-Carmona 2020 [OPEN-S-19] · Short	?	+	+	?	?	?
Márquez-Vera 2024 [OPEN-S-16] · Short	−	+	+	−	−	−
Packer 2015 [OPEN-S-20] · Short	+	+	+	?	?	?
Reeve 2024 [OPEN-S-21] · Short	+	+	?	+	+	?
Sancakli 2015 [OPEN-S-22] · Short	?	+	?	+	?	?
Topaloglu 2025 [OPEN-S-24] · Short	−	+	+	−	?	−
Zotelli 2017 [OPEN-S-25] · Short	?	+	+	?	?	?
Alajbeg I 2020 [OPEN-M-01] · Medium	?	+	?	+	?	?
de Oliveira 2024 [OPEN-M-02] · Medium	+	+	?	?	?	?
Gikic 2021 [OPEN-M-03] · Medium	?	+	?	?	?	?
Montes-Carmona 2020 [OPEN-M-04] · Medium	?	+	+	?	?	?

\+ Low   ? Some concerns   − High

D1 randomization; D2 deviations from intended interventions; D3 missing outcome data; D4 outcome measurement; D5 selection of the reported result.

RoB 2 traffic-light plot: Jaw functional limitation

Result-specific assessments (n=6); effect of assignment to intervention

Study / result	D1 Randomization	D2 Deviations	D3 Missing data	D4 Measurement	D5 Reporting	Overall
Calixtre 2019 [JFL-S-01] · Short	+	+	+	−	?	−
de Oliveira 2024 [JFL-S-02] · Short	+	+	?	?	?	?
de Salles Neto 2020 [JFL-S-03] · Short	+	+	?	+	?	?
Reeve 2024 [JFL-S-04] · Short	+	+	−	+	+	−
de Oliveira 2024 [JFL-M-01] · Medium	+	+	?	?	?	?
Gikic 2021 [JFL-M-02] · Medium	?	+	?	?	?	?

+ Low ? Some concerns − High

D1 randomization; D2 deviations from intended interventions; D3 missing outcome data; D4 outcome measurement; D5 selection of the reported result.

RoB 2 traffic-light plot: OHRQoL

Result-specific assessments (n=5); effect of assignment to intervention

Study / result	D1 Randomization	D2 Deviations	D3 Missing data	D4 Measurement	D5 Reporting	Overall
Chami 2022 [QOL-S-01] · Short	+	?	?	+	?	?
de Oliveira 2024 [QOL-S-02] · Short	+	+	?	?	?	?
de Salles Neto 2020 [QOL-S-03] · Short	+	+	?	+	?	?
de Oliveira 2024 [QOL-M-01] · Medium	+	+	?	?	?	?
Gikic 2021 [QOL-M-02] · Medium	?	+	?	?	?	?

+ Low ? Some concerns − High

D1 randomization; D2 deviations from intended interventions; D3 missing outcome data; D4 outcome measurement; D5 selection of the reported result.
