# Supplementary material for: Conservative and Minimally Invasive Treatments for Temporomandibular Disorders Versus Inactive Controls: A Systematic Review and Meta-Analysis of Randomized Trials
Source: Med Sci (Basel). 2026 Oct 10;14(6):660. doi: 10.3390/medsci14060660 (PMC13706717; doi:10.3390/medsci14060660)
Supplement: Supplementary file 1 [file medsci-14-00660-s001.zip › File_S2_PRISMA_2020_Checklist.pdf]

PRISMA 2020 Checklist

Conservative and Minimally Invasive Treatments for Temporomandibular Disorders versus Inactive Controls: A Systematic Review and Meta-Analysis of Randomized Trials

Locations refer to the submitted manuscript and accompanying supplementary materials.

Section and topic	Item #	Checklist item	Location where item is reported
**TITLE**			
Title	**1**	Identify the report as a systematic review.	p. 1, title.
**ABSTRACT**			
Abstract	**2**	See the PRISMA 2020 for Abstracts checklist.	p. 2, Abstract.
**INTRODUCTION**			
Rationale	**3**	Describe the rationale for the review in the context of existing knowledge.	pp. 2-3, Section 1, Introduction.
Objectives	**4**	Provide an explicit statement of the objective(s) or question(s) the review addresses.	p. 3, final paragraph of Section 1, Introduction.
**METHODS**			
Eligibility criteria	**5**	Specify the inclusion and exclusion criteria for the review and how studies were grouped for the syntheses.	p. 4, Section 2.2 and Table 1; p. 5, Section 2.5.
Information sources	**6**	Specify all databases, registers, websites, organisations, reference lists and other sources searched or consulted to identify studies. Specify the date when each source was last searched or consulted.	Partially reported: pp. 4–5, Section 2.3; Supplementary Table S1. The final database searches are reported as completed in January 2026; exact search dates for the individual sources are not reported.
Search strategy	**7**	Present the full search strategies for all databases, registers and websites, including any filters and limits used.	pp. 4–5, Section 2.3; Supplementary Table S1.
Selection process	**8**	Specify the methods used to decide whether a study met the inclusion criteria of the review, including how many reviewers screened each record and each report retrieved, whether they worked independently, and if applicable, details of automation tools used in the process.	p. 5, Section 2.4.
Data collection process	**9**	Specify the methods used to collect data from reports, including how many reviewers collected data from each report, whether they worked independently, any processes for obtaining or confirming data from study investigators, and if applicable, details of automation tools used in the process.	p. 5, Section 2.4.
Data items	**10a**	List and define all outcomes for which data were sought. Specify whether all results that were compatible with each outcome domain in each study were sought (e.g. for all measures, time points, analyses), and if not, the methods used to decide which results to collect.	pp. 4-5, Sections 2.2, 2.4 and 2.5; Table 1.
Data items	**10b**	List and define all other variables for which data were sought (e.g. participant and intervention characteristics, funding sources). Describe any assumptions made about any missing or unclear information.	p. 5, Section 2.4; pp. 9-10, Table 2.
Study risk of bias assessment	**11**	Specify the methods used to assess risk of bias in the included studies, including details of the tool(s) used, how many reviewers assessed each study and whether they worked independently, and if applicable, details of automation tools used in the process.	p. 6, Section 2.7.
Effect measures	**12**	Specify for each outcome the effect measure(s) (e.g. risk ratio, mean difference) used in the synthesis or presentation of results.	pp. 5-6, Sections 2.5 and 2.6.
Synthesis methods	**13a**	Describe the processes used to decide which studies were eligible for each synthesis (e.g. tabulating the study intervention characteristics and comparing against the planned groups for each synthesis (item #5)).	pp. 4-6, Sections 2.2 and 2.4-2.6; Table 1.
Synthesis methods	**13b**	Describe any methods required to prepare the data for presentation or synthesis, such as handling of missing summary statistics, or data conversions.	p. 5, Section 2.4.
Synthesis methods	**13c**	Describe any methods used to tabulate or visually display results of individual studies and syntheses.	p. 6, Section 2.6. Synthesis displays are presented in Figures 2–6 and Tables 2–5 (pp. 8–19); structured individual-study results are presented in Supplementary Table S4.
Synthesis methods	**13d**	Describe any methods used to synthesize results and provide a rationale for the choice(s). If meta-analysis was performed, describe the model(s), method(s) to identify the presence and extent of statistical heterogeneity, and software package(s) used.	pp. 5-6, Section 2.6; File S1 (R code).
Synthesis methods	**13e**	Describe any methods used to explore possible causes of heterogeneity among study results (e.g. subgroup analysis, meta-regression).	p. 6, Section 2.6.
Synthesis methods	**13f**	Describe any sensitivity analyses conducted to assess robustness of the synthesized results.	p. 6, Section 2.6.
Reporting bias assessment	**14**	Describe any methods used to assess risk of bias due to missing results in a synthesis (arising from reporting biases).	p. 6, Section 2.6.
Certainty assessment	**15**	Describe any methods used to assess certainty (or confidence) in the body of evidence for an outcome.	p. 6, Section 2.7.
**RESULTS**			

Section and topic	Item #	Checklist item	Location where item is reported
Study selection	16a	Describe the results of the search and selection process, from the number of records identified in the search to the number of studies included in the review, ideally using a flow diagram.	pp. 6-7, Section 3.1 and Figure 1.
Study selection	16b	Cite studies that might appear to meet the inclusion criteria, but which were excluded, and explain why they were excluded.	Not reported at report level. Figure 1 (p. 7) reports 314 full-text exclusions only in aggregate, without study-specific citations or reasons.
Study characteristics	17	Cite each included study and present its characteristics.	pp. 7–10, Section 3.2 and Table 2; all included studies are cited in references 57–91. Supplementary Table S4 links contributing studies or independent cohorts to outcomes, follow-up windows, interventions and inactive controls.
Risk of bias in studies	18	Present assessments of risk of bias for each included study.	p. 17, Section 3.4 and Figure 6; Supplementary Figures S2A and S2B; Supplementary Table S4 (result-specific overall judgments).
Results of individual studies	19	For all outcomes, present, for each study: (a) summary statistics for each group (where appropriate) and (b) an effect estimate and its precision (e.g. confidence/credible interval), ideally using structured tables or plots.	Supplementary Table S4 presents group sample sizes, means and standard deviations, effect estimates, standard errors and 95% confidence intervals for all 69 effects in the nine primary outcome-by-follow-up syntheses, plus the four arm-level contrasts used only in the exploratory treatment-cluster analysis. Figures 3 and 4 (pp. 12 and 14) display the two largest syntheses as forest plots.
Results of syntheses	20a	For each synthesis, briefly summarise the characteristics and risk of bias among contributing studies.	pp. 7–10, Section 3.2 and Table 2; p. 17, Section 3.4 and Figure 6; Supplementary Figure S2B; Supplementary Table S4.
Results of syntheses	20b	Present results of all statistical syntheses conducted. If meta-analysis was done, present for each the summary estimate and its precision (e.g. confidence/credible interval) and measures of statistical heterogeneity. If comparing groups, describe the direction of the effect.	pp. 11-19, Section 3.3, Figures 3-5 and Tables 3-5; Data S1.
Results of syntheses	20c	Present results of all investigations of possible causes of heterogeneity among study results.	pp. 11 and 13, comparator-stratified analyses; pp. 15-17, Sections 3.3.5 and 3.3.6.
Results of syntheses	20d	Present results of all sensitivity analyses conducted to assess the robustness of the synthesized results.	pp. 11, 13 and 15–17, Sections 3.3.1–3.3.3, 3.3.5 and 3.3.6.
Reporting biases	21	Present assessments of risk of bias due to missing results (arising from reporting biases) for each synthesis assessed.	pp. 17-18, Sections 3.3.6 and 3.5.
Certainty of evidence	22	Present assessments of certainty (or confidence) in the body of evidence for each outcome assessed.	pp. 18-19, Section 3.5 and Tables 4-5; Supplementary Table S3.
**DISCUSSION**			
Discussion	23a	Provide a general interpretation of the results in the context of other evidence.	pp. 19-24, Sections 4.1-4.7; pp. 25-26, Section 5.
Discussion	23b	Discuss any limitations of the evidence included in the review.	p. 24, Section 4.6; p. 25, Section 4.8.
Discussion	23c	Discuss any limitations of the review processes used.	p. 25, Section 4.8.
Discussion	23d	Discuss implications of the results for practice, policy, and future research.	pp. 24-25, Sections 4.7 and 4.9; pp. 25-26, Section 5.
**OTHER INFORMATION**			
Registration and protocol	24a	Provide registration information for the review, including register name and registration number, or state that the review was not registered.	p. 2, Abstract; pp. 3-4, Section 2.1; PROSPERO CRD420251250251.
Registration and protocol	24b	Indicate where the review protocol can be accessed, or state that a protocol was not prepared.	p. 3, Section 2.1; reference 27 (published protocol).
Registration and protocol	24c	Describe and explain any amendments to information provided at registration or in the protocol.	pp. 3-4, Section 2.1; p. 6, Section 2.8.
Support	25	Describe sources of financial or non-financial support for the review, and the role of the funders or sponsors in the review.	p. 26, Funding.
Competing interests	26	Declare any competing interests of review authors.	p. 26, Conflicts of Interest.
Availability of data, code and other materials	27	Report which of the following are publicly available and where they can be found: template data collection forms; data extracted from included studies; data used for all analyses; analytic code; any other materials used in the review.	p. 26, Data Availability Statement; Supplementary Table S4 (arm-level summary statistics and study-level effect estimates); Data S1 (analysis dataset); File S1 (R code). Additional extraction data are available from the corresponding author on reasonable request.

Source: Page MJ, McKenzie JE, Bossuyt PM, Boutron I, Hoffmann TC, Mulrow CD, et al. The PRISMA 2020 statement: an updated guideline for reporting systematic reviews. BMJ 2021;372:n71. doi:10.1136/bmj.n71. Checklist reproduced and completed under CC BY 4.0.
