# Supplementary material for: Conservative and Minimally Invasive Treatments for Temporomandibular Disorders Versus Inactive Controls: A Systematic Review and Meta-Analysis of Randomized Trials
Source: Med Sci (Basel). 2026 Oct 10;14(6):660. doi: 10.3390/medsci14060660 (PMC13706717; doi:10.3390/medsci14060660)
Supplement: Supplementary file 1 [file medsci-14-00660-s001.zip › Table_S1_Search_Strategies.pdf]

Supplementary Table S1. Database-Specific Search Strategies and Records Retrieved

Systematic review of conservative and minimally invasive interventions for temporomandibular disorders

Reporting note. Searches were last updated in January 2026. Strategies are reproduced as executed; only line breaks and spacing were added for readability. Counts are records retrieved before deduplication.

Search overview

Database	Platform / interface	Limits recorded in the executed strategy	Records
PubMed/MEDLINE	PubMed	English; 1 Jan 2015–31 Dec 2025; trial publication-type filters	104
Embase	Embase.com	English; article; publication years 2015–2025	143
Web of Science Core Collection	Web of Science	Article or Early Access; not Review Article; publication years 2015–2025	495
Scopus	Scopus	English; article; publication years 2015–2025; exact keyword ‘Randomized Controlled Trial’	373
Cochrane CENTRAL	Cochrane Library	English; publication years 2015–2025	19
**Total database records**	—	**Before deduplication**	**1,134**

1. PubMed/MEDLINE

Records retrieved: 104

Date and language limits: 1 January 2015–31 December 2025; English

Interface filters: Clinical Trial; Clinical Trial, Phase II; Clinical Trial, Phase III; Clinical Trial, Phase IV; Controlled Clinical Trial; Randomized Controlled Trial

Search strategy

```
( "temporomandibular joint disorders"[Mesh] OR "temporomandibular disorder*"[tiab] OR "temporomandibular joint dysfunction*"[tiab] OR "tmj disorder*"[tiab] OR "tmj dysfunction*"[tiab] OR "craniomandibular disorder*"[tiab] OR TMD[tiab] OR TMJ[tiab] )
AND
( "conservative treatment"[tiab] OR "conservative therapy"[tiab] OR "non-surgical"[tiab] OR "noninvasive"[tiab] OR "manual therapy"[tiab] OR physiotherapy[tiab] OR "physical therapy"[tiab] OR "exercise therapy"[tiab] OR rehabilitation[tiab] OR "occlusal splint"[tiab] OR "stabilization splint"[tiab] OR "oral appliance"[tiab] OR "anterior repositioning splint"[tiab] OR "laser therapy"[tiab] OR "low level laser"[tiab] OR photobiomodulation[tiab] OR TENS[tiab] OR ultrasound[tiab] OR "dry needling"[tiab] OR acupuncture[tiab] OR "platelet rich plasma"[tiab] OR PRP[tiab] OR "hyaluronic acid"[tiab] OR "botulinum toxin"[tiab] OR arthrocentesis[tiab] )
AND
( "quality of life"[tiab] OR wellbeing[tiab] OR OHIP[tiab] OR "oral health impact profile"[tiab] OR HRQoL[tiab] OR "health related quality of life"[tiab] OR "SF-36"[tiab] OR "SF-12"[tiab] OR "EQ-5D"[tiab] OR function[tiab] OR "jaw function"[tiab] OR "jaw movement"[tiab] OR "functional limitation"[tiab] OR disability[tiab] OR "psychological distress"[tiab] OR anxiety[tiab] OR depression[tiab] )
AND
```

```
( randomized[tiab] OR randomised[tiab] OR "randomized controlled trial"[pt] OR "controlled clinical trial"[pt] OR trial[tiab] OR controlled[tiab] )
NOT
( "Case Reports"[pt] OR "Review"[pt] OR "Systematic Review"[pt] OR "Meta-Analysis"[pt] OR "Editorial"[pt] OR "Comment"[pt] OR "Letter"[pt] OR protocol[tiab] ) AND ("2015/01/01"[dp] : "2025/12/31"[dp]) AND English[lang]
```

2. Embase

Records retrieved: 143

Limits: Publication years 2015–2025; English; article

Search strategy

```
('temporomandibular joint disorder'/exp OR 'temporomandibular disorder*':ti,ab OR 'temporomandibular joint dysfunction*':ti,ab OR 'tmj disorder*':ti,ab OR 'tmj dysfunction*':ti,ab OR 'craniomandibular disorder*':ti,ab OR tmd:ti,ab OR tmj:ti,ab) AND ('conservative treatment':ti,ab OR 'conservative therapy':ti,ab OR 'non-surgical':ti,ab OR noninvasive:ti,ab OR 'manual therapy':ti,ab OR physiotherapy:ti,ab OR 'physical therapy':ti,ab OR 'exercise therapy':ti,ab OR rehabilitation:ti,ab OR 'occlusal splint':ti,ab OR 'stabilization splint':ti,ab OR 'oral appliance':ti,ab OR 'anterior repositioning splint':ti,ab OR 'laser therapy':ti,ab OR 'low level laser':ti,ab OR photobiomodulation:ti,ab OR tens:ti,ab OR ultrasound:ti,ab OR 'dry needling':ti,ab OR acupuncture:ti,ab OR 'platelet rich plasma':ti,ab OR prp:ti,ab OR 'hyaluronic acid':ti,ab OR 'botulinum toxin':ti,ab OR arthrocentesis:ti,ab) AND ('quality of life':ti,ab OR wellbeing:ti,ab OR ohip:ti,ab OR 'oral health impact profile':ti,ab OR hrqol:ti,ab OR 'health related quality of life':ti,ab OR 'sf-36':ti,ab OR 'sf-12':ti,ab OR 'eq-5d':ti,ab OR function:ti,ab OR 'jaw function':ti,ab OR 'jaw movement':ti,ab OR 'functional limitation':ti,ab OR disability:ti,ab OR 'psychological distress':ti,ab OR anxiety:ti,ab OR depression:ti,ab) AND ('randomized controlled trial'/exp OR 'controlled clinical trial'/exp OR randomized:ti,ab OR randomised:ti,ab OR trial:ti,ab OR controlled:ti,ab) NOT ('case report'/exp OR 'review'/exp OR 'systematic review'/exp OR 'meta analysis'/exp OR editorial:pt OR letter:pt OR comment:pt OR protocol:ti,ab) AND [2015-2025]/py AND english:la AND article:it
```

3. Web of Science Core Collection

Records retrieved: 495

Publication-year filter: 2015–2025

Document-type filters: Article or Early Access; Review Article excluded

Search strategy

```
TS=( ("temporomandibular" OR "TMD" OR "TMJ") AND (disorder* OR dysfunction* OR arthralgia OR "disc displacement"))
AND TS=("randomized controlled trial" OR randomized OR randomly OR randomised OR "clinical trial" OR "controlled trial")
AND TS=(splint* OR "occlusal splint" OR "stabilization splint" OR "manual therapy" OR physiotherapy OR "physical therapy" OR "exercise therapy" OR photobiomodulation OR "low level laser" OR TENS OR ultrasound OR "dry needling" OR acupuncture OR "hyaluronic acid" OR PRP OR "platelet rich plasma" OR arthrocentesis OR "intra-articular injection")
NOT TS=("mechanism" OR "review" OR "systematic review" OR "guideline")
```

4. Scopus

Records retrieved: 373

Limits: Publication years 2015–2025; English; article; exact keyword ‘Randomized Controlled Trial’

Search strategy

```
TITLE-ABS-KEY ( "temporomandibular disorder*" OR "temporomandibular joint dysfunction*" OR "tmj disorder*" OR "tmj dysfunction*" OR "craniomandibular disorder*" OR TMD OR TMJ )
AND TITLE-ABS-KEY ( "conservative treatment" OR "conservative therapy" OR "manual therapy" OR physiotherapy OR "physical therapy" OR "exercise therapy" OR rehabilitation OR "occlusal splint" OR "stabilization splint" OR "oral appliance" OR "anterior repositioning splint" OR "laser therapy" OR "low level laser" OR photobiomodulation OR TENS OR ultrasound OR "dry needling" OR acupuncture OR "platelet rich plasma" OR PRP OR "hyaluronic acid" OR "botulinum toxin" OR arthrocentesis )
AND TITLE-ABS-KEY ( random* OR trial OR controlled OR comparative )
AND ( PUBYEAR > 2014 AND PUBYEAR < 2026 )
AND PUBYEAR > 2014 AND PUBYEAR < 2026
AND ( LIMIT-TO ( LANGUAGE , "English" ) )
AND ( LIMIT-TO ( DOCTYPE , "ar" ) )
AND ( LIMIT-TO ( EXACTKEYWORD , "Randomized Controlled Trial" ) )
```

5. Cochrane Central Register of Controlled Trials (CENTRAL)

Records retrieved: 19

Limits: Publication years 2015–2025; English

Search strategy

```
(((temporomandibular NEXT joint NEXT disorder*) OR (temporomandibular NEXT disorder*) OR (temporomandibular NEXT joint NEXT dysfunction*) OR (temporomandibular NEXT dysfunction*) OR (tmj NEXT disorder*) OR (tmj NEXT dysfunction*) OR (craniomandibular NEXT disorder*) OR TMD OR TMJ):ti,ab,kw)
AND
(("conservative treatment" OR "conservative therapy" OR "non-surgical" OR nonsurgical OR "non-invasive" OR noninvasive OR "manual therapy" OR physiotherapy OR "physical therapy" OR "exercise therapy" OR rehabilitation OR (occlusal NEXT splint*) OR (stabilization NEXT splint*) OR (oral NEXT appliance*) OR (anterior NEXT repositioning NEXT splint*) OR "laser therapy" OR (low NEXT level NEXT laser*) OR photobiomodulation OR TENS OR ultrasound OR "dry needling" OR acupuncture OR "platelet rich plasma" OR PRP OR "hyaluronic acid" OR (botulinum NEXT toxin) OR arthrocentesis OR ("intra-articular" NEXT injection*) OR (intraarticular NEXT injection*)):ti,ab,kw)
AND
(("quality of life" OR wellbeing OR OHIP OR "oral health impact profile" OR HRQoL OR "health related quality of life" OR "SF-36" OR "SF-12" OR "EQ-5D" OR function OR "jaw function" OR "jaw movement" OR "functional limitation" OR disability OR "psychological distress" OR anxiety OR depression):ti,ab,kw)
AND
((random):ti,ab,kw)
NOT
(protocol OR "study protocol" OR recruiting OR "not yet recruiting" OR "active, not recruiting" OR ongoing OR "no results available")
```

Abbreviations

CENTRAL, Cochrane Central Register of Controlled Trials; **HRQoL**, health-related quality of life; **OHIP**, Oral Health Impact Profile; **PRP**, platelet-rich plasma; **TENS**, transcutaneous electrical nerve stimulation; **TMD**, temporomandibular disorder; **TMJ**, temporomandibular joint.
