# Supplementary material for: Conservative and Minimally Invasive Treatments for Temporomandibular Disorders Versus Inactive Controls: A Systematic Review and Meta-Analysis of Randomized Trials
Source: Med Sci (Basel). 2026 Oct 10;14(6):660. doi: 10.3390/medsci14060660 (PMC13706717; doi:10.3390/medsci14060660)
Supplement: Supplementary file 1 [file medsci-14-00660-s001.zip › Table_S2_GRADEpro.pdf]

Author(s): Oana Țănculescu, Larisa Țarevici, Petronela Bodnar, Alice-Teodora Chiriac, Adrian Doloca, Vlad Ștefan Proca, Alina-Mihaela Apostu
Question: Conservative or minimally invasive treatments compared to Sham/placebo, no treatment/waitlist, or minimal/background care for Participants with temporomandibular disorders
Setting: Outpatient dental, oral medicine, maxillofacial, physiotherapy, and rehabilitation settings
Bibliography:

Certainty assessment							№ of patients		Effect		Certainty	Importance
№ of studies	Study design	Risk of bias	Inconsistency	Indirectness	Imprecision	Other considerations	Conservative or minimally invasive treatments	Sham/placebo, no treatment/waitlist, or minimal/background care	Relative (95% CI)	Absolute (95% CI)		
**Pain intensity — short term (follow-up: range 0 months to 3 months; assessed with: VAS/NRS pain scales harmonized to 0–10)**												
23	randomised trials	serious[a]	very serious[b]	not serious	serious[c]	none	676	545	-	MD **1.31 points lower** (2 lower to 0.62 lower)	⊕○○○ Very low[a,b,c]	CRITICAL
**Pain intensity — medium term (follow-up: range 5 months to 6 months; assessed with: VAS/NRS pain scales harmonized to 0–10)**												
4	randomised trials	serious[d]	not serious	not serious	serious[e]	none	113	60	-	MD **1.12 points lower** (2.02 lower to 0.23 lower)	⊕⊕○○ Low[d,e]	CRITICAL
**Pain intensity — long term (follow-up: mean 8 months; assessed with: VAS pain scale harmonized to 0–10)**												
1	randomised trials	very serious[f]	not serious	not serious	serious[g]	none	42	44	-	MD **4.35 points lower** (5.17 lower to 3.53 lower)	⊕○○○ Very low[f,g]	CRITICAL
**Mouth opening — short term (follow-up: range 0 months to 3 months; assessed with: Millimetric measurement of mandibular opening)**												
25	randomised trials	serious[h]	very serious[i]	serious[j]	serious[k]	none	768	572	-	MD **1.96 mm higher** (0.33 higher to 3.6 higher)	⊕○○○ Very low[h,i,j,k]	IMPORTANT
**Mouth opening — medium term (follow-up: range 5 months to 6 months; assessed with: Millimetric measurement of mandibular opening)**												
4	randomised trials	serious[l]	not serious	serious[m]	serious[n]	none	113	60	-	MD **2.25 mm higher** (1.6 lower to 6.1 higher)	⊕○○○ Very low[l,m,n]	IMPORTANT
**Jaw functional limitation — short term (follow-up: range 1 months to 3 months; assessed with: MFIQ, MOPDS, and JFLS-8)**												
4	randomised trials	serious[o]	serious[p]	not serious	very serious[q]	none	105	92	-	SMD **0.31 SD lower** (0.91 lower to 0.29 higher)	⊕○○○ Very low[o,p,q]	IMPORTANT
**Oral-health-related quality of life — short term (follow-up: range 1 months to 3 months; assessed with: OHIP/TMD and OHIP-14)**												
3	randomised trials	serious[r]	not serious	not serious	very serious[s]	none	62	42	-	SMD **0.17 SD lower** (1.04 lower to 0.7 higher)	⊕○○○ Very low[r,s]	IMPORTANT
**Jaw functional limitation — medium term (follow-up: range 5 months to 6 months; assessed with: MFIQ and JFLS)**												
2	randomised trials	serious[t]	not serious	not serious	very serious[u]	none	56	28	-	SMD **0.24 SD lower** (3.2 lower to 2.71 higher)	⊕○○○ Very low[t,u]	IMPORTANT
**Oral-health-related quality of life — medium term (follow-up: range 5 months to 6 months; assessed with: OHIP-14)**												
2	randomised trials	serious[v]	not serious	not serious	very serious[w]	none	56	28	-	MD **2.29 points lower** (35.89 lower to 31.3 higher)	⊕○○○ Very low[v,w]	IMPORTANT

CI: confidence interval; **MD:** mean difference; **SMD:** standardised mean difference

Explanations

a. Downgraded one level for risk of bias: 10 of 23 results were at high risk of bias and the remaining 13 had some concerns; none was at low risk. Excluding the high-risk results attenuated the pooled mean difference from −1.31 to −0.94 points.
b. Downgraded two levels for inconsistency: heterogeneity was considerable (I^2 = 93.6%), and τ = 1.50 points exceeded the 1-point clinical threshold. The 95% prediction interval ranged from −4.51 to 1.89 points, spanning important benefit, no effect, and possible harm. Prespecified moderator analyses did not explain this variability.
c. Downgraded one level for imprecision: the 95% confidence interval ranged from −2.00 to −0.62 points and crossed the prespecified 1-point clinical threshold. The estimate is therefore compatible with both a clinically unimportant and a clinically important average benefit.
d. Downgraded one level for risk of bias: one of four results was at high risk and the remaining three had some concerns; none was at low risk. Removing the high-risk result changed the confidence interval from excluding no effect to including no effect
e. Downgraded one level for imprecision: the 95% confidence interval ranged from −2.02 to −0.23 points and crossed the prespecified 1-point clinical threshold. The sensitivity analysis excluding the high-risk result also included no effect.
f. Downgraded two levels for risk of bias because the entire body of evidence consists of one study judged at high risk of bias. No result at low risk or with only some concerns and no independent replication were available.
g. Downgraded one level for imprecision. Although the confidence interval lies entirely beyond the 1-point clinical threshold, the analyzed sample of 86 participants did not meet the optimal information size: approximately 117 participants at 80% power or 157 participants at 90% power, based on a pooled SD of 1.93 and a 1-point target difference.
h. Downgraded one level for risk of bias: 9 of 26 results were at high risk and the remaining 17 had some concerns; none was at low risk. Excluding the high-risk results reduced the pooled estimate from 1.96 to 0.75 mm, and the confidence interval then included no effect.
i. Downgraded two levels for inconsistency: heterogeneity was considerable (I^2 = 85.9%), and τ = 3.51 mm approximated the 3.5-mm clinical threshold. The 95% prediction interval ranged from −5.46 to 9.39 mm, spanning important harm, no effect, and important benefit.
j. Downgraded one level for indirectness because the synthesis combined pain-free, maximum unassisted, assisted, and incompletely specified mouth-opening measurements. These constructs are clinically related but not interchangeable.
k. Downgraded one level for imprecision: the 95% confidence interval ranged from 0.33 to 3.60 mm and crossed the prespecified 3.5-mm clinical threshold. The sensitivity analysis excluding high-risk results also included no effect.
l. Downgraded one level for risk of bias because all four results had some concerns and none was at low risk of bias.
m. Downgraded one level for indirectness because mouth-opening constructs and stopping or assistance criteria were not uniform across studies, limiting inference for any single clinical opening measure.
n. Downgraded one level for imprecision: the 95% confidence interval ranged from 1.60 mm lower to 6.10 mm higher. It crossed no effect and the 3.5-mm threshold for clinically important benefit, but did not reach the corresponding threshold for clinically important harm.
o. Downgraded one level for risk of bias: two of four results were at high risk and the other two had some concerns; none was at low risk. The body of evidence remaining after exclusion of high-risk results contained only two studies and was extremely imprecise.
p. Downgraded one level for inconsistency: individual effects ranged from −0.73 to 0.08, and τ = 0.23 standardized units exceeded the prespecified 0.20 threshold. The prediction interval included clinically important effects in both directions.
q. Downgraded two levels for imprecision: the 95% confidence interval ranged from −0.91 to 0.29 standardized units, crossed no effect, and included effects beyond the 0.20 threshold in both the beneficial and harmful directions. Only 197 participants from four studies contributed.
r. Downgraded one level for risk of bias because all three results had some concerns and none was at low risk of bias.
s. Downgraded two levels for imprecision: the 95% confidence interval ranged from −1.04 to 0.70 standardized units, crossed no effect, and included clinically important benefit and harm relative to the prespecified 0.20 threshold. Only 104 participants from three studies contributed.
t. Downgraded one level for risk of bias because both results had some concerns and neither was at low risk of bias.
u. Downgraded two levels for imprecision: the 95% confidence interval ranged from −3.20 to 2.71 standardized units and included very large effects in both directions relative to the prespecified 0.20 threshold. Only 84 participants from two studies contributed.
v. Downgraded one level for risk of bias because both results had some concerns and neither was at low risk of bias.
w. Downgraded two levels for imprecision: the 95% confidence interval ranged from −35.89 to 31.30 OHIP-14 points and included very large benefit and harm relative to the prespecified 5-point threshold. Only 84 participants from two studies contributed.
